# Synergistic intravesical instillation for bladder cancer: CRISPR-Cas13a and fenbendazole combination therapy

**DOI:** 10.1186/s13046-024-03146-0

**Published:** 2024-08-12

**Authors:** Mingkang Liang, Yongqiang Wang, Lisha Liu, Dashi Deng, Zeqin Yan, Lida Feng, Chenfan Kong, Chenchen Li, Yuqing Li, Guangzhi Li

**Affiliations:** 1 Institute of Urology, The Affiliated Luohu Hospital of Shenzhen University, Shenzhen University, Shenzhen, Guangdong 518000 China; 2 Institute of Urology, Luohu Clinical College of Shantou University Medical College, Shantou University Medical College, Shantou, Guangdong 515000 China; 3 Department of Urology, Health Science Center, South China Hospital, Shenzhen University, Shenzhen, Guangdomg 518116 China

**Keywords:** CRISPR-Cas13a, Flubendazole, Fluorinated chitosan, Bladder cancer, Programmed death ligand 1, Transmembrane peptides

## Abstract

**Background:**

CRISPR-Cas13a is renowned for its precise and potent RNA editing capabilities in cancer therapy. While various material systems have demonstrated efficacy in supporting CRISPR-Cas13a to execute cellular functions in vitro efficiently and specifically, the development of CRISPR-Cas13a-based therapeutic agents for intravesical instillation in bladder cancer (BCa) remains unexplored.

**Methods:**

In this study, we introduce a CRISPR-Cas13a nanoplatform, which effectively inhibits PDL1 expression following intravesical instillation. This system utilizes a fusion protein CAST, created through the genetic fusion of CRISPR-Cas13 and the transmembrane peptide TAT. CAST acts as a potent transmembrane RNA editor and is assembled with the transepithelial delivery carrier fluorinated chitosan (FCS). Upon intravesical administration into the bladder, the CAST-crRNAa/FCS nanoparticles (NPs) exhibit remarkable transepithelial capabilities, significantly suppressing PDL1 expression in tumor tissues.To augment immune activation within the tumor microenvironment, we integrated a fenbendazole (FBZ) intravesical system (FBZ@BSA/FCS NPs). This system is formulated through BSA encapsulation followed by FCS coating, positioning FBZ as a powerful chemo-immunological agent.

**Results:**

In an orthotropic BCa model, the FBZ@BSA/FCS NPs demonstrated pronounced tumor cell apoptosis, synergistically reduced PDL1 expression, and restructured the immune microenvironment. This culminated in an enhanced synergistic intravesical instillation approach for BCa. Consequently, our study unveils a novel RNA editor nanoagent formulation and proposes a potential synergistic therapeutic strategy. This approach significantly bolsters therapeutic efficacy, holding promise for the clinical translation of CRISPR-Cas13-based cancer perfusion treatments.

**Supplementary Information:**

The online version contains supplementary material available at 10.1186/s13046-024-03146-0.

## Background

Bladder cancer (BCa) is the tenth most commonly diagnosed cancer globally, marked by high recurrence and progression rates [1, 2]. Intravesical instillation-based chemotherapy [3] or Bacillus Calmette-Guérin (BCG) immunotherapy, primarily used after transurethral resection of bladder tumors (TURBT), is vital in eliminating residual tumor cells and preventing BCa recurrence or progression [4]. Conventional chemotherapeutics such as mitomycin [5, 6], epirubicin [7, 8], and gemcitabine [9, 10], alongside the classical immunomodulator BCG [11], currently stand as first-line treatments for BCa perfusion therapy. Recently, checkpoint-targeted immunotherapeutics (e.g., Avelumab, Pembrolizumab, and Durvalumab) have been incorporated into BCa immunotherapy as standalone treatments or combined with neoadjuvant chemotherapy [12–17]. However, the efficacy of intravesical instillation and immunotherapy remains suboptimal, hindered by limited bioavailability [18] and the immunosuppressive microenvironment of bladder tumors. This gap underscores the urgent need for innovative transepithelial platforms and effective agents to enhance immune activation and improve BCa treatment outcomes.

Gene therapy, aimed initially at managing genetic disorder phenotypes, has increasingly focused on cancer therapy [19]. To some extent, the therapy makes up for the shortcomings of traditional therapies for bladder cancer, for example, the use of novel bacterial immunotherapy in the bladder induces rejection of non-responsive bladder tumors with BCG vaccine [20–22]. Mannose-binding protein FimH imparts mannose-targeting capabilities to improve BCG immunotherapy for bladder cance [23]. CRISPR-Cas13a, a preeminent gene-editing tool, has demonstrated promise in malignant tumor gene therapy. The Cas13a-CRISPR-derived RNA(crRNA) complex, known for its high specificity in targeting and cleaving single-stranded RNA from tumor cells [24], offers an alternative approach for various tumor treatments [25, 26]. The development of CRISPR-Cas13a in gene therapy has spurred extensive research into novel delivery methods, such as nano-cocoons and liposome systems, to improve its cellular uptake [27, 28]. Futhermore,the human immunodeficiency virus (HIV) transcriptional activator (TAT) can be used as a delivery functional substance, and the long TAT protein is chemically cross-linked with a variety of different proteins, such as horseradish peroxidase, β-galactosidase, etc. It can be transfected into cultured cells without the need for a receptor.However, studies on Cas13a-crRNA systems specifically tailored for intravesical BCa treatment are scarce. Additionally, immunotherapy's effectiveness is often compromised by tumor immunosuppression. While targeting immune checkpoints can significantly bolster immune regulation [29, 30], limited immune cell infiltration remains a substantial barrier to therapeutic success.The combination with other therapies is an effective way to promote the infiltration of immune cells. Many studies have shown that the combination of gene therapy and chemotherapy can effectively improve the tumor suppression limitations of single gene therapy. Therefore, there is an urgent need to develop chemotherapeutic drugs that can inhibit immune checkpoint, increase immune infiltration and have tumor killing effect. Fenbendazole (FBZ), traditionally used as a broad-spectrum anthelmintic [31], has recently emerged as an effective antitumor agent, influencing ferroptosis, autophagy, mitotic mutation, apoptosis, and cell cycle arrest in various cancer cells [32–37]. Its role as a novel PD-1 inhibitor and immunologic adjuvant has shown promise in enhancing the antitumor activity of immune cells, offering a new direction in BCa immunotherapy [38, 39]. However, FBZ's hydrophobic nature and non-selective cytotoxicity to cancer cells, coupled with low bioavailability in perfusion, pose significant challenges for its intravesical administration in BCa. These limitations impede effective tumor-targeted transepithelial delivery, constraining FBZ's therapeutic potential in intravesical instillation for BCa. The perfusion application and mechanism of action of FBZ in BCa treatment remain underexplored.

In our study, a fusion protein combining transmembrane peptide TAT and Cas13a was synthesized through genetic engineering and aimed to bind with PDL-1crRNAa. This Cas13a-TAT-crRNA complex (CAST-crRNAa) demonstrated enhanced cellular uptake and reduced PDL1 expression in tumor cells. Building on our previous work with fluorinated chitosan (FCS), a potent transepithelial delivery carrier, we coated CAST-crRNAa with FCS, forming Cas13a-TAT-crRNA/FCS nanoparticles (CAST-crRNAa/FCS NPs). When administered intravesically, the interaction with FCS facilitates transepithelial delivery of CAST-crRNAa, rearranging tight junction proteins in bladder epithelium and enabling TAT-mediated cellular internalization and PDL1 expression regulation in tumor tissues.

**Scheme 1:**
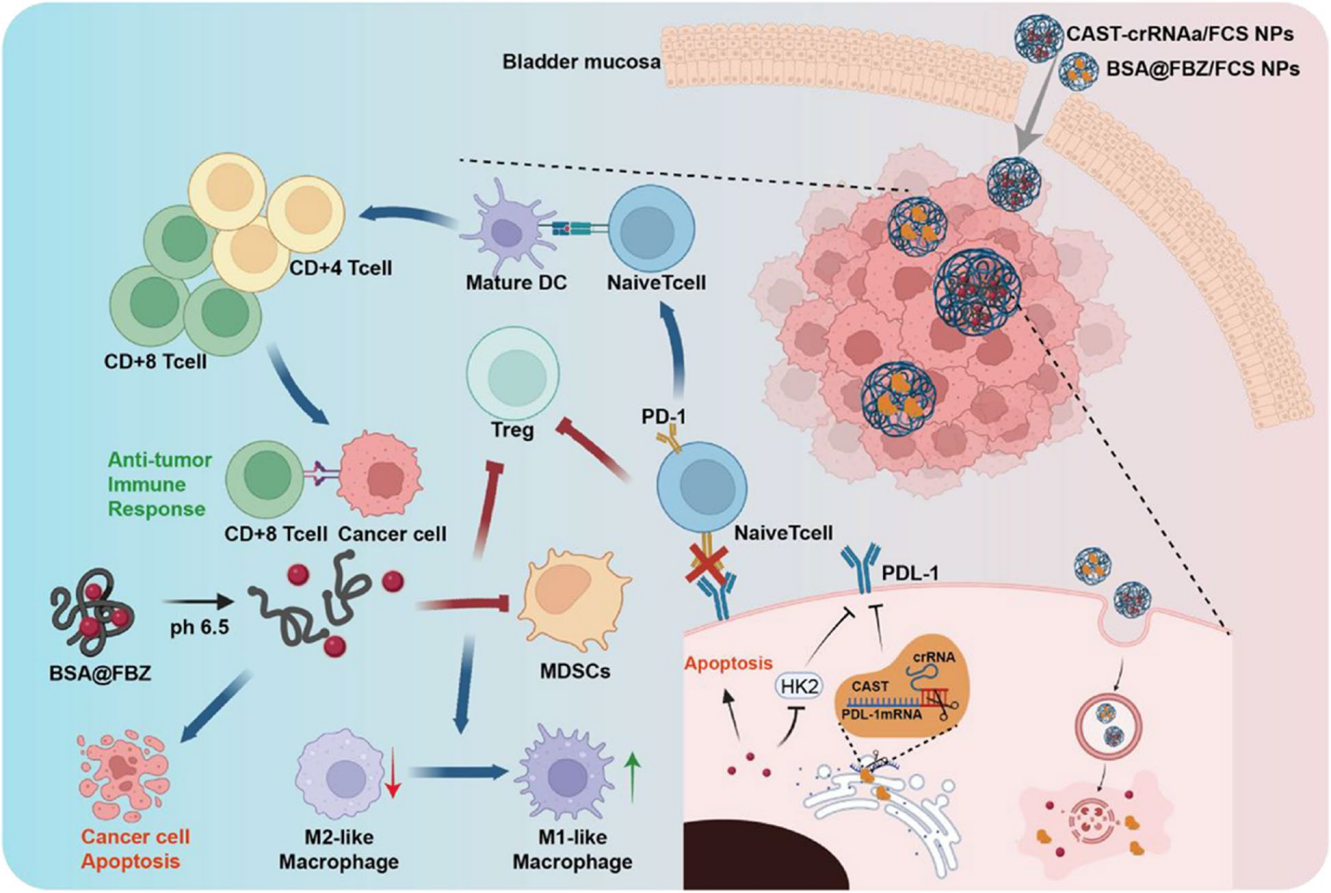
.

We developed a well-suited FBZ intravesical formulation with efficient transepithelial tumor-targeting capability to further address the tumor's immunosuppressive environment. In this formulation, FBZ was encapsulated in BSA, leveraging disulfide bond cleavage and condensation properties, and then combined with FCS to create BSA@FBZ/FCS NPs. This significantly enhanced FBZ's perfusion bioavailability. In acidic and high H_2_O_2_ tumor tissue conditions, a charge reversal effect and FBZ release from BSA@FBZ were induced, respectively. Our findings indicate that BSA@FBZ/FCS NPs effectively induce apoptosis and down-regulate programmed death-ligand1 (PDL-1) in tumor cells, through influencing hexokinase2 (HK2) expression in the endoplasmic reticulum. In vivo, the combined application of CAST-crRNA/FCS NPs and BSA@FBZ/FCS NPs markedly downregulated PDL1 expression with negligible off-target effect and reshaped the immune microenvironment in tumor tissues, significantly inhibiting BCa tumor growth and prolonging mouse survival (scheme 1).

## Results and discussion

### Preparation and characterization of CAST-crRNA/FCS NPs and BSA@FBZ/FCS NPs

**Fig. 1:**
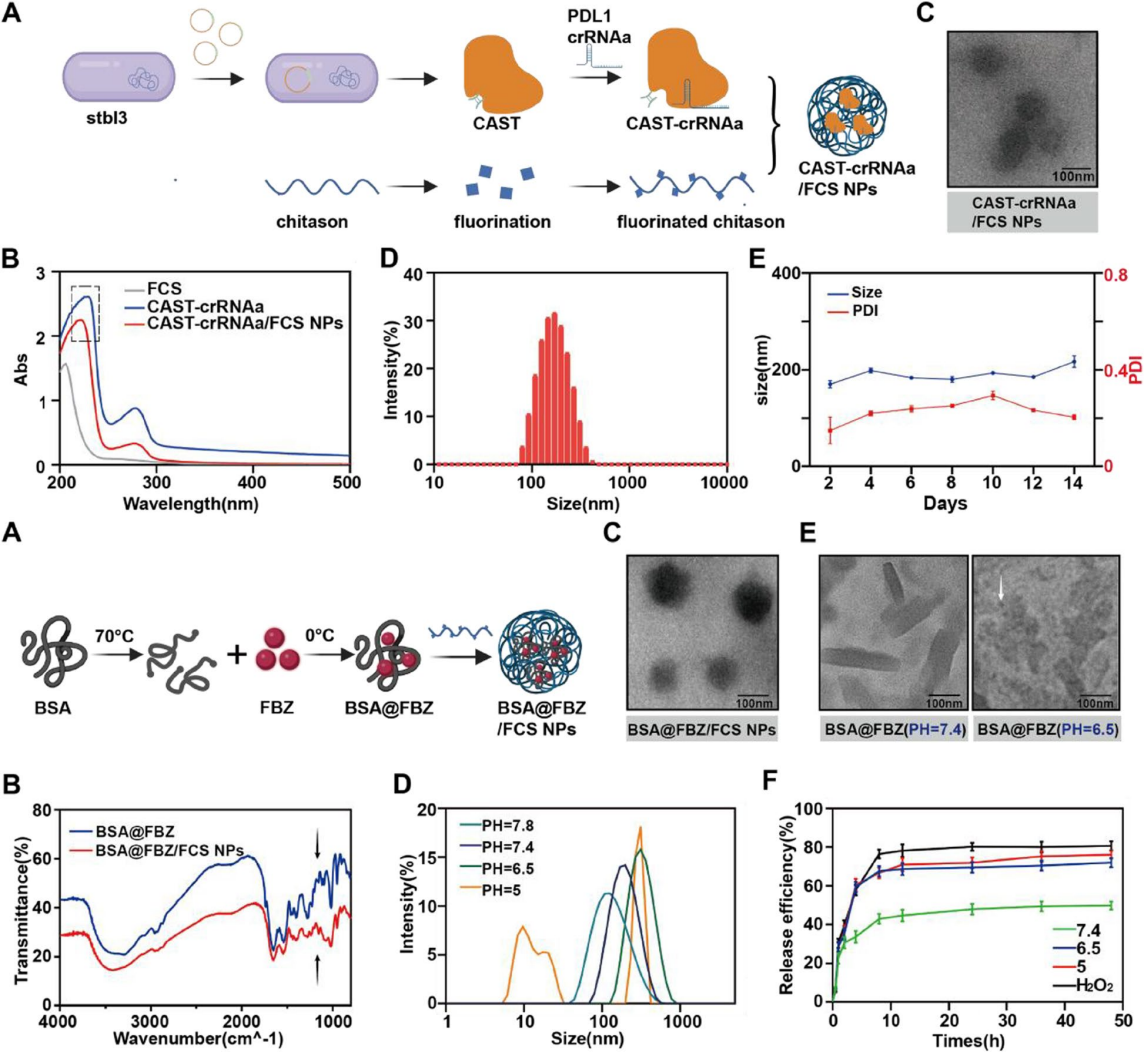
Preparation and characterization of CAST-crRNAa/FCS NPs and BSA@FBZ/FCS NPs. **A** Simple synthesis diagram of CAST-crRNAa/FCS NPs. **B** UV spectrophotometer spectra of FCS, CAST-crRNAa, and CAST-crRNAa/FCS NPs. **C** Transmission electron microscope(TEM) image and **D** Hydrodynamic diameters of CAST-crRNAa/FCS NPs. **E** Changes in CAST-crRNAa/FCS NPs hydrodynamic diameter and PDI values within 14 days. **F** Simple synthesis diagram of BSA@FBZ/FCS NPs. **G** Fourier Transform Infrared Spectroscopy(FTIR) of BSA@FBZ and BSA@FBZ/FCS NPs. **H** TEM of BSA@FBZ/FCS NPs. **I** Distribution of hydrodynamic diameter of BSA@FBZ under different pH values (5.0~7.8). **J** TEM images of BSA@FBZ under pH 7.4 and 6.5. **K** The release profiles of BSA@FBZ/FCS NPs at pH 7.4, 6.5, 5.0 or H2O2 within 48 hours

Following a previously established procedure, FCS was synthesized by grafting perfluorohexane onto chitosan (CS) [40, 41]. The CAST protein was produced by purifying competent cells co-expressing plasmids containing Cas13a and TAT (refer to Figs. 1A, S1, and S2). Western blotting confirmed CAST's protein specificity for Cas13a, which is evidenced by a slightly higher molecular weight (see Figure S3). Subsequently, the most effective PDL1 crRNA targeting PDL1 mRNA was identified using the TaqMan method. Real-time quantitative PCR (qPCR) evaluated the relative PDL1 mRNA levels in MB49 cells treated with CAST-crRNAa, CAST-crRNAb, crRNAa, CAST, or Phosphate Buffer Solution (PBS). Both TaqMan and qPCR analyses indicated that CAST-crRNAa was the most efficient in specific recognition and cleavage of PDL1 mRNA (Figures S4, S5).

The formation of CAST-crRNA/FCS NPs involved an electrostatic reaction between FCS and CAST-crRNAa. UV–visible absorbance spectra characterized the various CAST-crRNAa/FCS NP formulations. A shift in the characteristic peak of CAST-crRNAa from 240 to 225 nm signified successful assembly with FCS (Fig. 1B). Coomassie Brilliant Blue staining determined the optimal CAST-crRNAa to FCS ratio. Grayscale value analysis demonstrated maximum encapsulation efficiency at a mass ratio of 1:4 (Figures S6, S7). Transmission electron microscopy (TEM) revealed that CAST-crRNAa/FCS NPs possessed a highly dispersed uniform spherical morphology, with a hydrodynamic diameter of approximately 170 ± 7.53 nm and a zeta potential of approximately 16.71 ± 0.32 mV, as measured by Zetasizer Lab (Figs. 1C, D). The CAST-crRNAa/FCS NP solution exhibited no significant fluctuations in particle size or PDI values over 14 d (Fig. 1E), indicating excellent stability in aqueous solutions. Hydrodynamic diameter analysis and Polydispersity Index (PDI) were used to evaluate the stability of CAST-crRNAa/FCS NPs at different temperatures. As shown in the Figure S13, the hydrodynamic diameter and PI of NPs did not change significantly at 4, 25, 37℃,but increased at 70℃(Figure S13).

To synthesize BSA@FBZ/FCS, a solution of BSA in water at 70 °C and FBZ in DMSO was mixed thoroughly and then rapidly cooled, followed by centrifugation to yield the supernatant (BSA@FBZ). This supernatant was assembled with FCS to form BSA@FBZ/FCS NPs (Fig. 1F). High-performance liquid chromatography (HPLC) determined the drug loading of BSA@FBZ to be approximately 11.0 ± 2.7%. The hydrodynamic size of the BSA@FBZ to FCS mixture, at a weight ratio of 1:1, was around 182.7 ± 1.52 nm, with a zeta potential of 13.47 ± 1.27 mV (Figure S8). Fourier transform infrared spectroscopy (FTIR) analysis demonstrated changes in the absorption peak at 1200 cm^−1^, indicating successful binding of BSA@FBZ and FCS (Fig. 1G). TEM imaging revealed that BSA@FBZ/FCS NPs had a highly dispersed, uniform, spherical morphology (Fig. 1H).

In contrast, BSA-FBZ/FCS NPs were synthesized using a well-established approach, wherein FBZ was loaded through hydrophobic interactions instead of the disulfide bond reconstruction in BSA. As Figure S9 demonstrates, BSA-FBZ had significantly lower drug encapsulation efficiency than BSA@FBZ. Additionally, the BSA-FBZ and BSA-FBZ/FCS NPs exhibited increased and non-uniform hydrodynamic diameters compared to the BSA@FBZ and BSA@FBZ/FCS NPs groups (Figure S10). These findings suggest that the BSA@FBZ/FCS NPs, with their superior drug-loading capacity and dispersibility, are more suitable for this study.

The pH-sensitive and reduction-responsive characteristics of BSA@FBZ/FCS NPs were evaluated by examining morphological changes, size fluctuations, charge inversion, and drug release performance under low pH and high H_2_O_2_ conditions. As illustrated in Figure S11, the zeta potential of the NPs shifted from -20.57 ± 1.65 mV to 19.67 ± 0.41 mV when the solution's pH was gradually lowered from 7.4 to 6.5. Additionally, a noticeable expansion in size distribution and collapse in morphology were observed (Fig. 1I, J). The FBZ release profiles from the BSA@FBZ/FCS NPs at different pH values (5.0, 6.5, and 7.4) and in high H_2_O_2_ concentrations (10 mM) were analyzed (Fig. 1K). At PBS (pH 7.4), only 49.9 ± 2.1% of FBZ was released within 48 h, which significantly increased under low pH (pH 6.5, 72.0 ± 2.3%; pH 5.0, 76.1 ± 2.0%) and high H_2_O_2_ conditions (80.7 ± 2.3%). The stability of BSA@FBZ/FCS NPs in PBS was assessed by monitoring size distribution and PDI over 14 d. As Figure S12 indicates, the NPs showed no significant fluctuations in size or PDI, demonstrating the notable stability of the BSA@FBZ/FCS NPs solution. However, the stability of the NPs decreased at high temperature(Figure S13).

### Transepithelial delivery of nanoparticles across bladder epithelium

**Fig. 2:**
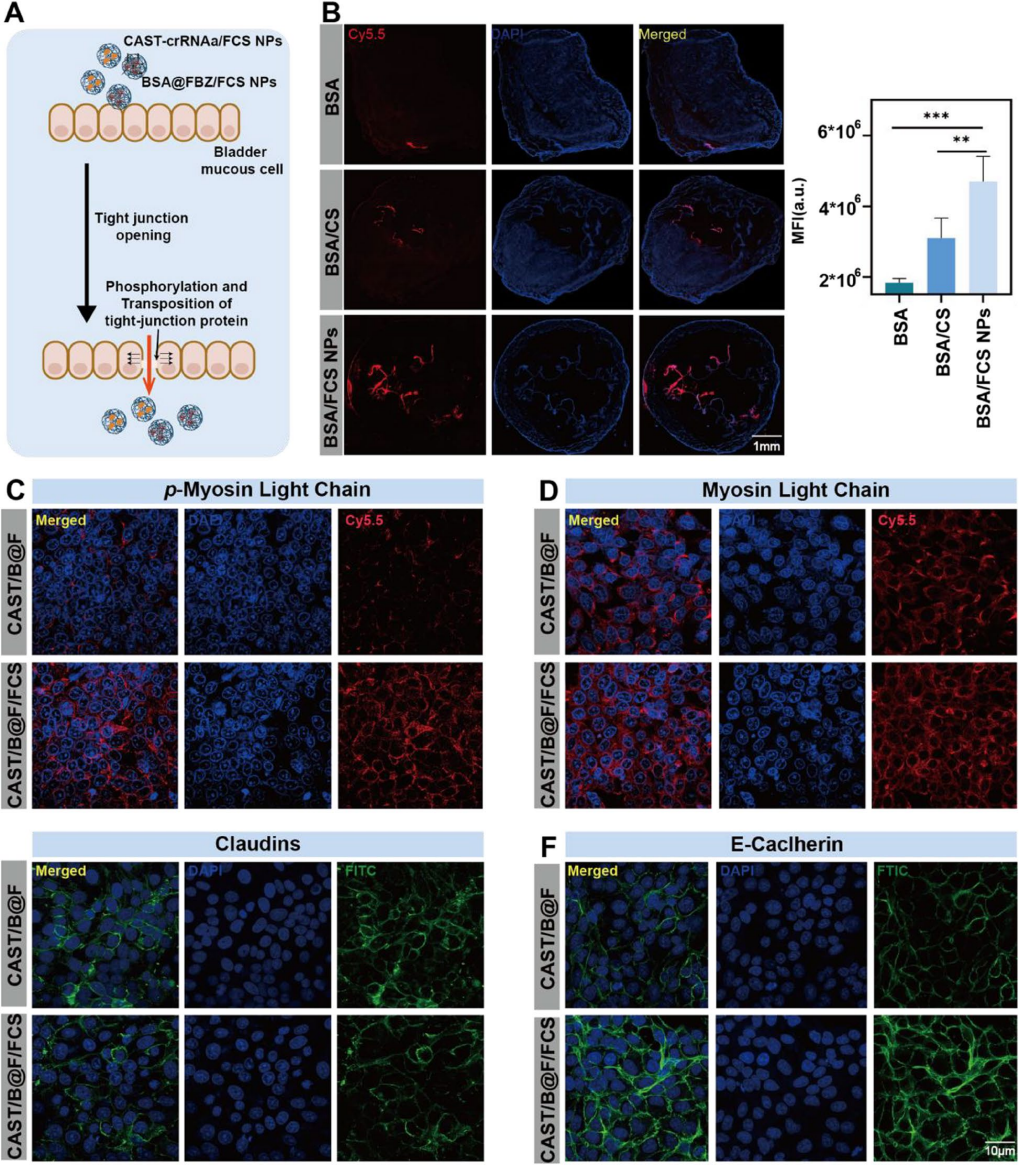
Transepithelial delivery of BSA/FCS NPs in bladder tissues and the mechanism of transepithelial action of CAST/B@F/FCS. **A** Schematic illustration of transepithelial delivery of CAST/B@F/FCS NPs and BSA@FBZ/FCS NPs. **B** Confocal Laser Scanning Microscopy (CLSM) images of fluorescence localization of BSA in bladder cancer tissues under treatment of BSA/FCS NPs, BSA/CS NPs, free BSA. Statistical analysis of mean fluorescence intensity (MFI) using ZEISS software. **C-F** CLSM images of cellular tight junction proteins P-myosin light chain (MLC), E-cadherin, MLC and Claudin in SV-HUC-1 cells. (Data are means ± SD.Statistical significance was determined by unpaired two-tailed Student’s t-test ( **P < 0.01, ***P < 0.001))

The fluorine chains within the nanoparticles (NPs) facilitate the partial phosphorylation and transposition of epithelial tight junction proteins. This process effectively opens cellular gaps, aiding in the transepithelial transport of intravesical agents (Fig. 2A). To evaluate the tumor transepithelial capacity of these NPs, BSA was tagged with cyanine 5.5. Mice with orthotopic BCa tumors received intravesical instillation of free BSA/FCS NPs, BSA/CS, or free BSA (2 mg/kg) for 1 h before bladder harvest. Confocal laser scanning microscopy (CLSM) results (Fig. 2B) demonstrated that mice treated with BSA/FCS NPs exhibited significantly stronger BSA fluorescence in vertical bladder slices compared to the other groups, highlighting the exceptional transepithelial absorption ability of FCS NPs.To further explore the half-life of NPs in the bladder, BSA/FCS NPs were injected into the bladder, and the retention time of NPs in the bladder was observed using the biological in vivo imaging system. As shown in the Figure, the observation period was 16h, and the half-life is calculated by using the standard curve composed of fluorescence values at each time point, approximately 6.5 h(Figure S14).

Further investigation involved treating immortalized human ureteral epithelial cells (SV-HUC-1) with either CAST/B@F/FCS (CAST-crRNAa/FCS NPs + BSA@FBZ/FCS NPs) or CAST/FBZ (CAST-crRNAa + BSA@FBZ) for 24 h. CLSM analysis revealed morphological changes in tight junction proteins, including myosin light chain (MLC), p-MLC, claudins, and E-cadherin. Post-treatment with CAST/B@F/FCS claudins, and E-cadherin displayed blurred or discontinuous patterns, starkly contrasting the continuous ring-like features in the CAST/B@F group. Moreover, while the quantity of MLC in the CAST/B@F/FCS NPs group remained unchanged, the phosphorylated portion of MLC significantly increased (Figs. 2C-F). These observations align with our previous studies on the mechanism of transepithelial FCS, indicating that phosphorylation of MLC leads to the displacement of tight junction proteins, thereby facilitating the opening of tight junctions.

### Cellular uptake and lysosomal escape of CAST-crRNAa/FCS NPs and BSA@FBZ/FCS NPs

**Fig. 3:**
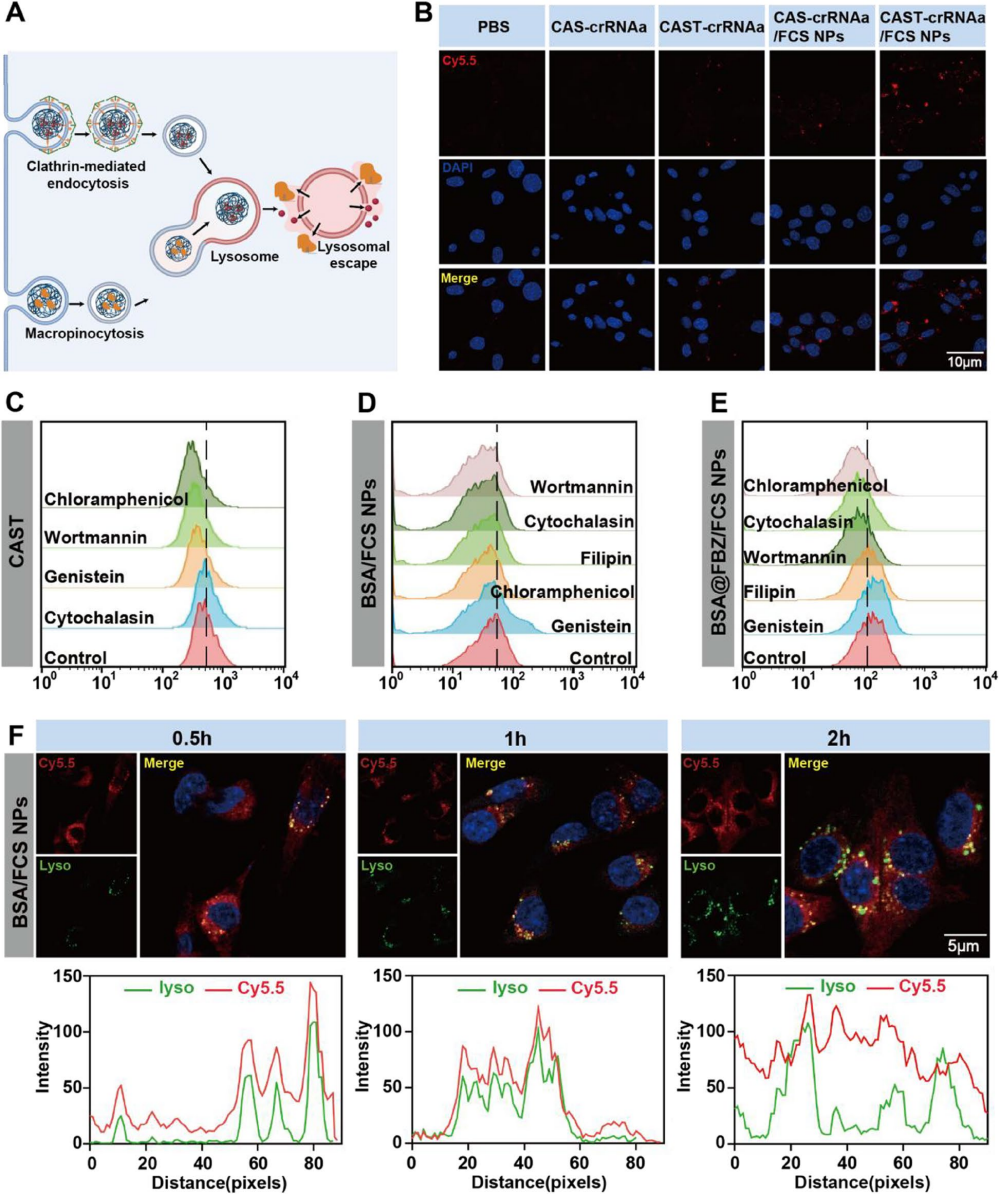
Cellular uptake, endocytic pathways, and lysosomal escape of CAST-crRNAa/FCS NPs and BSA@FBZ/FCS NPs. **A** Schematic illustration of cellular uptake, endocytic pathways, and lysosomal escape of CAST-crRNAa/FCS NPs and BSA@FBZ/FCS NPs. **B** CLSM images of cellular uptake of CAST-crRNAa/FCS NPs. **C-E** Flow cytometry detection of CAST-crRNAa, Cy5.5-BSA/FCS or Cy5.5-BSA@FBZ/FCS in MB49 cells under treatment with different endocytic inhibitors. **F** Localization of BSA/FCS NPs (markers, red) in MB49 cells at lysosome (markers,green) was assessed by CLSM image analysis

The effective functioning of drugs and proteins within cells necessitates their uptake and subsequent escape from lysosomes (Fig. 3A). To assess the synergistic impact of TAT and FCS on cellular uptake**,** we co-incubated MB49 cells with CAST-crRNAa/FCS NPs, CAS-crRNAa/FCS NPs, CAST-crRNAa, CAS-crRNAa, and PBS for 2 h. CLSM images and statistical analysis (Fig. 3B, S15) indicated a significantly higher fluorescence intensity in cells treated with CAST-crRNAa/FCS NPs and CAS-crRNAa/FCS NPs, compared to those with CAS-crRNAa and PBS. This finding suggests the enhancement of cellular uptake by TAT and FCS.

Further, to investigate the cellular uptake mechanisms, MB49 cells were pre-treated with various endocytic inhibitors (Chlorpromazine, Wortmannin, Genistein, Filipin, and cytochalasin) for 30 min before co-incubation with CAST-crRNAa, BSA/FCS NPs, or BSA@FBZ/FCS NPs for 2 h. Flow cytometry results post-chlorpromazine or wortmannin treatment demonstrated a significant decrease in fluorescence intensity in the CAST-crRNAa group (Fig. 3C). For BSA, the most pronounced uptake inhibition was observed with Wortmannin or cytochalasin (Fig. 3D), while for BSA@FBZ, the most significant inhibition occurred with Chlorpromazine and cytochalasin (Fig. 3E). These outcomes suggest that TAT primarily enhances Cas13a protein uptake via clathrin-dependent and macropinocytosis pathways. In contrast, FCS predominantly facilitates BSA uptake through macropinocytosis or phagocytosis and BSA@FBZ uptake via clathrin-dependent and macropinocytosis pathways.

Regarding the lysosomal escape of FCS NPs, previous studies suggest that FCS acts as a 'protective shield' for proteins and drugs against the acidic lysosomal environment. To verify this, MB49 cells were co-cultured with BSA/FCS NPs, BSA, BSA@FBZ/FCS NPs, and BSA@FBZ, and lysosomes were stained with lysotracker probes at intervals of 0.5, 1, 2, and 4 h. Results depicted in Figs. 3F, S16, and S17 demonstrated that in the BSA/FCS NPs group, BSA co-localized with lysosomes at 1 h and separated by 2 h, whereas in the BSA group, co-localization persisted at 2 h. A similar pattern was observed in the BSA@FBZ/FCS NPs and BSA@FBZ groups, corroborating our previous findings.

### The in vitro therapeutic effect and antitumor mechanism of CAST-crRNAa/FCS NPs and BSA@FBZ/FCS NPs

**Fig. 4:**
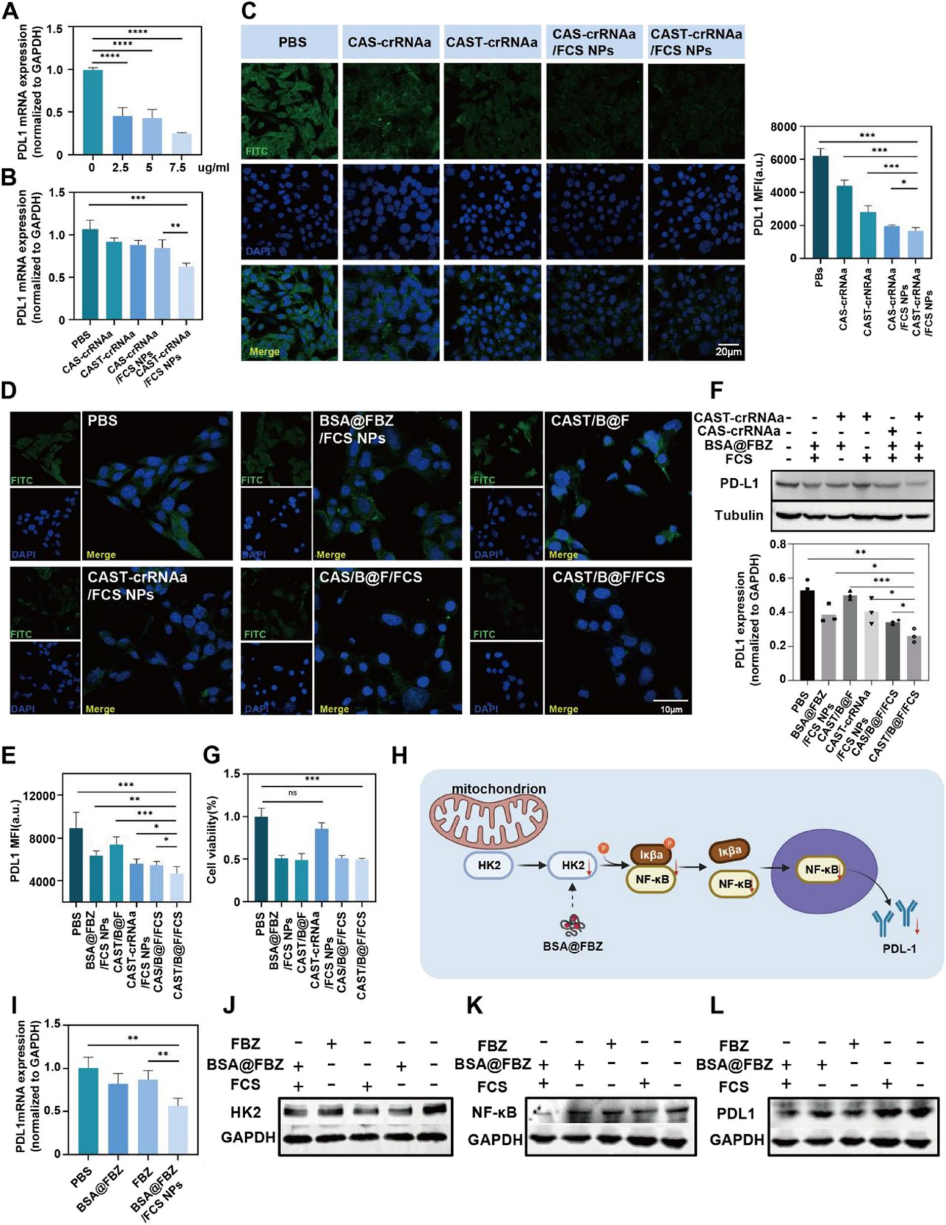
Therapeutic Effect and Antitumor Mechanism in Vitro of CAST-crRNAa/FCS NPs and BSA@FBZ/FCS NPs. **A** Real-time Quantitative PCR Detecting System(QPCR) detection of PDL-1 mRNA in MB49 cells treated by different concentrations (0, 2.5, 5, 7.5 ug/ml)of CAST-crRNAa/FCS. **B** QPCR detection of PDL1 mRNA in MB49 cells under PBS, CAS-crRNAa, CSAT-crRNAa, CAS-crRNAa/FCS NPs or CAST-crRNAa/FCS NPs treatments. **C** CLSM images of PDL-1 fluorescence in MB49 cells under PBS, CAS-crRNAa, CAST-crRNAa, CAS-crRNAa/FCS NPs, CAST-crRNAa/FCS NPs treatments.(left). Statistical analysis of mean fluorescence intensity (MFI) using ZEISS software (right). **D-E** CLSM images of PDL-1 protein in MB49 cells under PBS, BSA@FBZ/FCS NPs, CAST/FBZ, CAST-crRNAa/FCS NPs, CAS/B@F/FCS, CAST/B@F/FCS treatment. Statistical analysis of MFI using ZEISS software. **F** Western blot detection of PDL-1 protein in MB49 cells under different treatments. Quantitative analysis of western blot results using IMAGE J. **G** Statistical analysis of proliferation of MB49 cells under different treatment groups. **H** Schematic illustration of downregulation of tumor cell PDL1 protein by BSA@FBZ. **I** Detection of PDL1 mRNA under BSA@FBZ/FCS NPs, BSA@FBZ, FBZ, PBS treatments using QPCR. **J-K** Western blot detection of HK2, NF-kB, and PDL-1 expression in MB49 cells under BSA@FBZ/FCS NPs, BSA@FBZ, FBZ, FCS, PBS treatments. Quantitative analysis of western blot results using IMAGE J. (Data are means ± SD. Statistical significance was determined by unpaired two-tailed Student ‘ s t-test ( *P < 0.05, **P < 0.01, ***P < 0.001, ****P < 0.0001, ns, nonsignificant)

The Cas13a system, known for its ability to cleave target RNA, can indiscriminately cut any free single-stranded RNA once the CRISPR-Cas13 system forms a ternary complex with crRNA and the substrate. To identify the optimal concentration of CAST-crRNAa/FCS NPs for therapeutic use, Quantitative PCR (qPCR) were employed to measure the relative expression levels of PDL-1 mRNA in MB49 cells treated with varying concentrations of CAST-crRNAa/FCS NPs. As Fig. 4A illustrates, there was a gradual decrease in PDL-1 mRNA content with increasing concentrations of CAST-crRNAa/FCS NPs. Notably, at a concentration of 7.5 µg/mL, cells displayed significant fragmentation and blurred edges. Therefore, the viability of MB49 cells at different concentrations of CAST were studied. As shown in the Figure S18, cell viability decreased when the concentration of CAST was above 6ug/ml, while there was no significant difference at 4ug/ml. Additionally, the viability of normal urothelial SV-HUC1 cells was not affected (Figure S18).

Further, qPCR analysis revealed a substantial reduction in PDL-1 mRNA in cells treated with CAST-crRNAa/FCS NPs compared to other groups. Besides, only 3 genes were recorded to have significantly down-regulated by transcriptome-wide mRNA sequencing (Figure S19). These results indicate that CAST-crRNAa/FCS NPs significantly enhance the efficiency of PDL-1 mRNA cleavage without significant off-target effect, demonstrating their potential as a therapeutic agent. The observed dose-dependent effect underscores the need for precise concentration control to maximize therapeutic benefits while minimizing cytotoxicity, a critical factor in developing effective cancer treatments.

The augmentation of the Cas13a system by TAT and FCS modification is evident in the enhanced efficiency of PDL-1 mRNA cleavage (Fig. 4B). CLSM and fluorescence quantitative analysis further corroborated the most pronounced knockout of PDL-1 protein in the CAST-crRNAa/FCS NPs group (Fig. 4C). To assess the cytotoxic effects of combined therapy with CAST-crRNAa/FCS NPs and BSA@FBZ/FCS NPs, MB49 cells were treated with various formulations: PBS, BSA@FBZ/FCS NPs, CAST/B@F (CAST-crRNAa + BSA@FBZ), CAST-crRNAa/FCS NPs, CAS/B@F/FCS (CAS-crRNAa/FCS NPs + BSA@FBZ/FCS NPs), and CAST/B@F/FCS (CAST-crRNAa/FCS NPs + BSA@FBZ/FCS NPs). The treatments were applied for 24 h at specific concentrations (CAST-crRNAa, 5 µg/mL; BSA@FBZ, 1 µg/mL), and the subsequent expression of PDL-1 in cells was detected via CLSM.

Interestingly, the combination of CASt-crRNAa/FCS NPs and BSA@FBZ/FCS NPs resulted in a significant downregulation of PDL-1 expression in MB49 cells compared to the CAST-crRNAa/FCS NPs group alone (Fig. 4D, E). Western blot analysis, utilizing GAPDH as an internal control, revealed varying degrees of PDL1 downregulation by both CAST-crRNAa/FCS NPs and BSA@FBZ/FCS NPs. However, the combination therapy, CAST/B@F/FCS, demonstrated the most substantial downregulation of PDL-1 (Fig. 4F).In addition, MB49 cells and human bladder cancer cells (T24) were co-incubated in each treatment group, flow cytometry analysis showed that the expression of PDL1 in MB49 and T24 was significantly inhibited by the combination group(Figure S2^0^, S21). PDL1siRNA was designed according to the literature and combined with FCS to form nanoparticle siRNA/FCS NPs. Flow cytometry results showed that the knockout efficiency of Cas13a at safe concentrations was only 10% lower than that of siRNA (Figure S22).

In summary**,** BSA@FBZ/FCS NPs emerge as novel PDL1 inhibitors for bladder cancer immunotherapy, offering synergistic inhibition of immune checkpoints alongside CAST-crRNAa/FCS NPs, thereby reshaping the immune microenvironment. Moreover, BSA@FBZ/FCS NPs significantly inhibited MB49 tumor cell proliferation by promoting apoptosis. As Fig. 4G illustrates, cell activity was markedly reduced following treatment with CAST/B@F/FCS, CAS/B@F/FCS, CAST/B@F, or BSA@FBZ/FCS NPs, while the activity in the CAST-crRNAa/FCS NPs group was not significantly affected. Furthermore, flow cytometry results confirmed the substantial pro-apoptotic effect of BSA@FBZ/FCS NPs (Figure S23), highlighting their dual functionality in chemotherapy and immunostimulation.Additionally, BSA@FBZ/FCS and SV-HUC1 cells of different concentrations were cultured and cell viability were determined to investigate the toxic effect of BSA@FBZ/FCS on normal cells. As shown in the Figure S24, BSA@FBZ/FCS of different concentrations had no effect on the cell viability of SV-HUC1.

### Mechanisms of BSA@FBZ/FCS-Mediated PDL-1 down-regulation

**Fig. 5:**
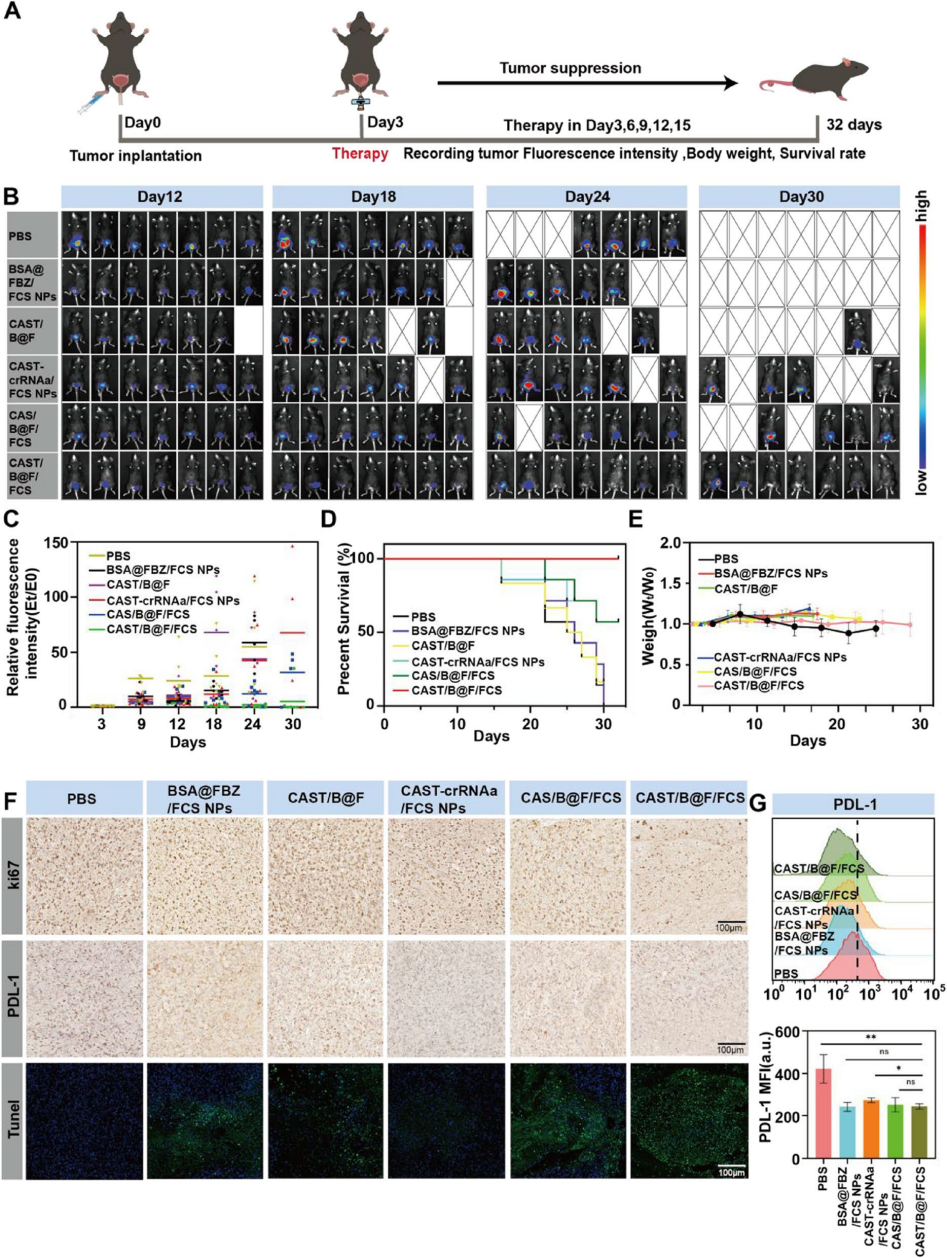
Synergistic therapy of the combination administration of CAST-crRNAa/FCS and BSA@FBZ/FCS. **A** Schematic of in vivo treatment of bladder tumors. **B** Aniview software-generated bioluminescent images of tumors in mice on Days 12, 18, 24, and 30. **C**. Statistical analysis of fluorescent signal values in tumors of mice using Aniview software from day 3 to day 30. **D** Survival analysis of mice in each group within 32 days. **E** Changes in body weight (Wt/W0) of mice in each group over four weeks. **F** Immunohistochemical images of Ki67, PDL-1 and Tunel. **G** Flow cytometry detection of PDL-1 in tumors of mice tumor from each groups. (Data are means ± SD.Statistical significance was determined by unpaired two-tailed Student’s t-test ( *P < 0.05, **P < 0.01, ns, nonsignificant).)

The influence of glycolytic enzymes within tumor cells, particularly hexokinase 2 (HK2), on the expression of tumor proteins through downstream pathways is well-established [42]. On the whole, HK2 isolated from mitochondria can act as a protein kinase to phosphorylate IKBa, resulting in IkBa degradation and NF-kB activation-dependent PD-L1 expression for tumor immune evasion. [43]. To explore the impact of BSA@FBZ/FCS NPs on HK2 and the mechanism of PDL1 downregulation, MB49 cells were treated with BSA@FBZ/FCS NPs, BSA@FBZ, free FBZ, or PBS, and the relative PDL-1 mRNA levels were assessed by qPCR. The findings revealed a significant downregulation of PDL-1 mRNA following treatment with FBZ-containing agents such as BSA@FBZ/FCS NPs, BSA@FBZ, and free FBZ (Fig. 4I). Western blot analysis further confirmed that, compared to FCS and PBS treatments, BSA@FBZ/FCS, BSA@FBZ, and FBZ notably down-regulated HK2, NF-κB, p-IkBa and PDL-1 proteins in MB49 cells (Figs. 5J, K, S25, S26). These results suggest that BSA@FBZ/FCS NPs effectively reduce HK2 levels in tumor cells, thereby inhibiting the NF-κB dependent PDL1 up-regulation pathway (Fig. 4H).

### Synergistic therapy of the combination of CAST-crRNAa/FCS NPs and BSA@FBZ/FCS NPs

Mice with orthotopic firefly-luciferase-expressing MB49 (fLuc-MB49) bladder tumors were randomly divided into six groups: PBS, B@FFCS NPs, CAST/B@F, CAST-crRNAa, CASTFCS NPs, CAS/B@F/FCS NPs, and CAST/B@F/FCS NPs and intravesically administration every three days (Fig. 5A). Following five intravesical instillations (Fig. 5B, C), the CAST/B@F/FCS group exhibited the most substantial tumor inhibition effect among all the groups. Furthermore, the survival rate of mice in the CAST/B@F/FCS group was notably prolonged (Fig. 5D).A recognized PDL1 inhibitor was then injected intravenously as a control group, compared with the CAST/B@F/FCS group. As shown in the Figure S27, both the combined use of the two nanoplatforms and the control group of intravenous PDL1 showed significant tumor inhibition. However, as the treatment cycle progressed, the PDL1 group of mice gradually died and lost weight, which may be due to the biological toxicity of PDL1.

To evaluate the biosafety of prolonged treatment with CAST-crRNAa/FCS NPs and BSA@FBZ/FCS NPs, we closely monitored changes in the body weight of mice throughout the observation period. Figure 5E illustrates that the body weight of mice remained stable following treatment with CAST/B@F/FCS. Additionally, an examination of vital organs (heart, liver, spleen, lung, and kidney) using hematoxylin and eosin (H&E) staining revealed no significant alterations in tissue structure, cellular morphology, or immune cell infiltration after intravesical injection of CAST/B@F/FCS. This indicates the excellent biosafety profiles of both CAST-crRNAa/FCS NPs and BSA@FBZ/FCS NPs, as further supported by the data in Figure S28.

Subsequent analyses included PDL-1, Ki-67 staining, Caspase3 staining and TUNEL assay of tumor sections from the treatment groups. As illustrated in Fig. 5F, S29, the CAST/B@F/FCS-treated group exhibited the lowest PDL-1 expression, evidenced by the fewest brown nuclei in the IHC staining for PDL-1. This group also displayed the lowest proliferative index, as indicated by the minimal brown nuclei in Ki-67 IHC staining, and the highest apoptotic index, characterized by maxmal brown nuclei in Caspase3 IHC staining and green staining in the TUNEL images, compared to all other groups. Further assessment using flow cytometry to measure PDL-1 expression within the tumors corroborated these findings, revealing a marked reduction in PDL-1 expression across all groups except the PBS group, as depicted in Fig. 5G. In additional, siRNA/FCS NPs Knock-out efficiency was less than CAST/B@F/FCS, only 39%, which may be related to the low stability and easy degradation of siRNA (Figure S30). These findings suggest that CAST/B@F/FCS inhibits tumor growth by downregulating PDL1 expression and promoting apoptosis in tumor cells.

### CAST/B@F/FCS Reshaped the tumor immunosuppression microenvironment

**Fig. 6:**
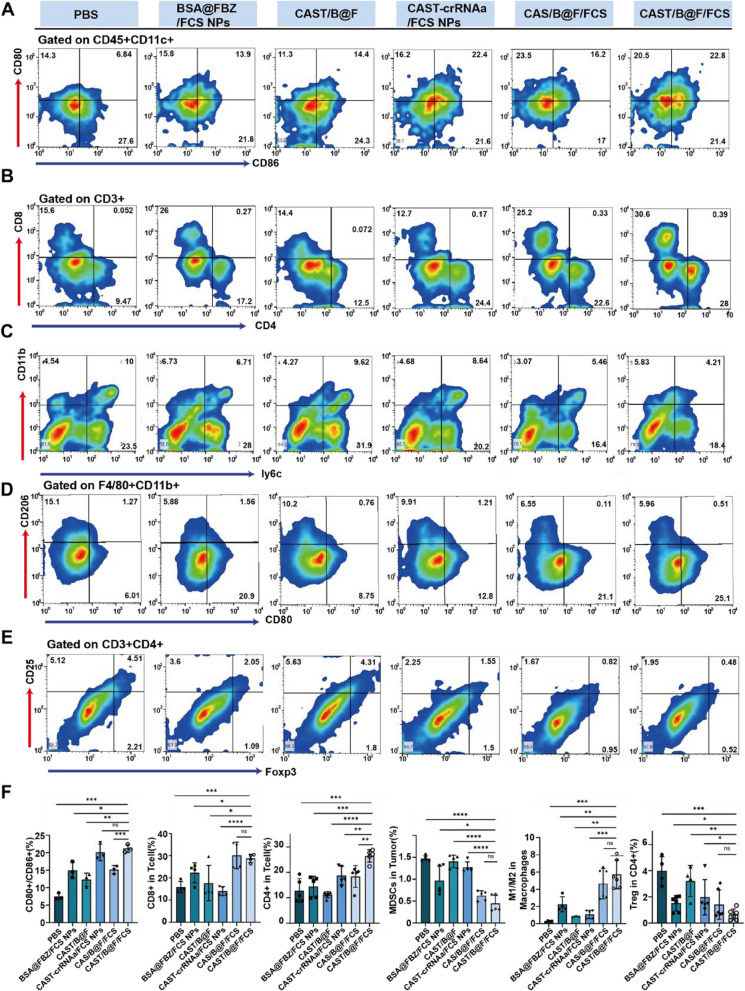
Assessment of Postive Immune Cell and Negative Immune Cell Levels in Mouse Tumors. **A-E** Flow cytometry analysis of intratumoral levels of Dendritic cells (DCs), T-lymphocyte cells, Myeloid-derived suppressor cells (MDSCs), Tumor-Associated Macrophages(TAM) and Regulatory cells(Tregs) in different therapy groups of mice. **F** Statistical analysis using Graphprism software for average values of immune cells in each groups. (Data are means ± SD.Statistical significance was determined by unpaired two-tailed Student’ s t-test ( *P < 0.05, **P < 0.01, ***P < 0.001, ****P < 0.0001, ns, nonsignificant.)

As expected, the mice treated with CAST/B@F/FCS demonstrated the highest expression of CD45 + CD11c + gated in CD80 + CD86 + DCs, as indicated in Fig. 6A and F. This finding suggests a predominance of mature DCs in this treatment group. The enhanced antigen-presenting ability of DCs plays a pivotal role in alleviating T-cell inhibition by cancer cells within the tumor microenvironment, thereby facilitating rapid T-cell activation. As depicted in Fig. 6B and F, treatment with CAST/B@F/FCS resulted in a significant increase in T-cell populations, with approximately 28.8 ± 1.88% CD3 + CD8 + T cells and 26.44 ± 2.44% CD3 + CD4 + T cells in the tumor, a notable rise compared to the PBS-treated groups.Furthermore, Positive cytokines such as TNF-a and IFN-y, which are related to T cell function, are also significantly upregulated(Figure S31).

Myeloid-derived suppressor cells (MDSCs), known for their role in tumor immune suppression, were also evaluated. These cells obstruct T cell-mediated antitumor immune responses, contribute to the polarization of M1-like macrophages into immunosuppressive M2-like macrophages, and assist in recruiting Treg cells [44]. Post-treatment with CAST/B@F/FCS and control treatments, the MDSC population within tumors was assessed. The data, illustrated in Fig. 6C and F, revealed a decrease in MDSCs from 1.47 ± 0.06% to 0.97 ± 0.33% following BSA@FBZ/FCS NPs treatment. A further reduction to 0.45 ± 0.19% in the MDSC population was observed in the group treated with CAST/B@F/FCS, attributed to the influence of BSA@FBZ/FCS NPs. Additionally, CD86 and CD206 markers, identified in CD11b + F4/80 + cells, were employed to differentiate between M1-like and M2-like macrophages within the tumor tissue. A significant increase in the M1/M2 macrophage ratio was observed following CAST/B@F/FCS treatment, as compared to other groups, as illustrated in Fig. 6D and F. Furthermore, Treg cells (CD25 + Foxp3 + gated in CD3 + CD4 + T cells), known to suppress the antitumor immune response, were analyzed. The application of CAST/B@F/FCS, CAS/B@F/FCS, CAST-crRNAa/FCS NPs, and BSA@FBZ/FCS NPs led to a marked reduction in the Treg cell population compared to other treatments, as illustrated in Fig. 6E and F.

These results indicate that BSA@FBZ/FCS NPs alone or CAST-crRNAa/FCS NPs can inhibit PDL1 expression to down-regulate the immunosuppressive effect in tumor to a certain extent, and the down-regulating effect is significantly amplified when the two are used in combination. The proapoptotic effect of BSA@FBZ/FCS NPs was also observed. In summary, the administration of CAST/B@F/FCS effectively remodeled the tumor immune microenvironment. This was achieved by promoting proapoptotic, enhancing the positive regulation of the immune response, suppressing its negative regulation, and amplifying the overall antitumor effectiveness immuneinfiltration.

## Conclusion

Our study successfully developed CAST-crRNAa and BSA@FBZ, which were self-assembled into CAST-crRNAa/FCS and BSA@FBZ/FCS NPs using the transepithelial delivery carrier FCS. This approach facilitated efficient transepithelial delivery and enhanced tumor cell uptake. Combining CAST-crRNAa/FCS NPs with BSA@FBZ/FCS NPs effectively inhibited PDL-1 expression in tumors. Moreover, it significantly upregulated positive immune cell populations and suppressed negative immune cells, reshaping the tumor immunosuppressive microenvironment and effectively curtailing the growth of bladder cancer.

Our findings revealed no significant side effects following perfusion therapy with either CAST-crRNAa/FCS NPs or BSA@FBZ/FCS NPs. Consequently, this study presents a novel strategy for administering bladder perfusion therapy using gene editing tools and delves into the mechanism underlying flubendazole's impact on bladder cancer therapy. This could hold substantial therapeutic value, particularly in suppressing postoperative residual tumor lesions and combating muscle-invasive bladder cancer.

## Methods

### Materials and animals

Chitosan (DD% ≥ 95%) was procured from Aladdin Industrial Co., Shanghai, China. N-(3-(Dimethylamino) propyl)-N-ethylcarbodiimide hydrochloride crystalline (EDC) and N-hydroxysuccinimide (NH) were sourced from JK Chemical Company, Beijing, China. Phosphate-buffered saline (PBS) was acquired from Beijing Solarbio Science & Technology Co. Ltd. DMEM/Ham’s F-12 medium, RPMI-1640 medium, fetal bovine serum, and penicillin/streptomycin were obtained from Life Technologies, New York, NY, USA. C57L mice were procured from Gempharmatech Co. Ltd.

### Construction, transfection, sequencing, purification, and characterization of plasmids

The selected plasmid was Escherichia coli plasmid pC013-Twinstrep-SUMO-huLwCas13a, featuring the TAT sequence GGACGTAAAAAGCGICGCCAACGGC GACGCCCACAGGGAICCATG. The plasmid was initially digested using BamHI (1 µl) restriction enzyme in a 40 µl system at 37 °C for 2 h. The TAT sequence was ligated to the digested product in a 10 µl system. Post-ligation, the product was introduced into STBL3 competent cells, chilled on ice for 30 min, heat-activated at 42 °C for 90 s, subsequently chilled for 2 min, transferred to LB medium containing AMP, shaken at 37 °C for 45 min, plated on solid LB medium, and incubated at 37 °C for 24 h. Following successful colony growth, selected colonies were sent to Shenzhen Kangti Life Technology Co., Ltd. for sequencing and purification, yielding Cas13a-TAT.

Subsequently, 10 µg each of Cas13a-TAT and Cas13a were extracted and added to an appropriate loading buffer. The samples were heated at 95 °C for 5 min, cooled, and then loaded onto an SDS-PAGE gel. Electrophoresis was performed at 80 V for 60 min. The gel was then transferred onto a PVDF membrane at 300 mA for 60 min. The membrane was blocked with skimmed milk for 1 h, incubated with a Cas13a primary antibody (1:1000, R&D Systems) overnight at 4 °C, washed thrice with TBST, incubated with an HRP-conjugated mouse secondary antibody (1:10,000, Affinity) for 60 min, washed thrice with TBST, and exposed to a 1:1 ratio of developing solution (A:B) for visualization.

CRISPR-derived RNA (crRNA) screening involved amplifying and purifying PDL1crRNA(a-c) and PDL-1 RNA templates. Using Cas13-TAT, crRNA(a-c), templates, assay buffer, DEPC water, RNAase inhibitor, and a fluorescent probe, a qPCR instrument was employed to collect the emitted signal from the probe. The CAST-crRNAa, CAST-crRNAb, pure crRNAa, pure CAST, and PBS groups were subsequently designed. Following co-incubation with MB49 cells for 16 h, RNA was extracted using a kit from Nanjing Vazyme Biotech Co., Ltd. Reverse transcription was performed to obtain cDNA using a reverse transcription kit from Nanjing Vazyme Biotech Co., Ltd. GAPDH (Guangzhou IGE Biotechnology Ltd.) served as an internal reference for qPCR system preparation. Each sample of the PDL-1crRNaA full length, consisting of 67 nucleotides (5'-3'):gggGAUUUAGACUACCCCAAAAACGAAGGGGACUAAAACcgcccUagcaagUgacagcaggcUgUg.

qGAPDH-F-M: CATCACTGCCACCCAGAAGACTG.

qGAPDH-R-M: ATGCCAGTGAGCT TCCCGTTCAG.

qPDL1-F-M: TGCGGACTACAAGCGAATCACG.

qPDL1-R-M: CTCAGCTTCTGGATAACCCTCG.

(GUANGZHOU IGE BIOTECHNOLOGY LTD).

### Preparation and characterization of BSA@FBZ and BSA-FBZ

BSA:FBZ was prepared at a 20:1 molar ratio. Initially, BSA (66 mg) was dissolved in 10 ml of DEPC water, while FBZ (12 mg) was dissolved in 1 ml of DMSO. The aqueous solution of BSA was heated at 70 °C for 15 min, after which the FBZ solution was added to the BSA solution before cooling. Vigorous shaking was employed for thorough mixing, followed by rapid cooling in an ice bath. Following cooling, the mixture was centrifuged at 5000 rpm for 5 min, and the supernatant was collected. HPLC analysis was conducted to determine the concentration and content of FBZ in the supernatants. FBZ exhibited a UV absorption peak around 254 nm, which facilitated the calculation of drug loading using the standard curve equation y = 20,055.97x + 10,217,702.37. The drug loading formula was defined as the mass of FBZ in the sample over the mass of freeze-dried powder.

The preparation of BSA-FBZ was similar to that of BSA@FBZ, except for the absence of heating, with direct mixing followed by centrifugation. The BSA@FBZ samples were immersed in solutions at pH levels 7.8, 7.4, 6.5, and 5.0 for 30 min to evaluate their particle sizes and zeta potentials. Additionally, BSA@FBZ in a pH 6.5 aqueous solution was applied onto a copper mesh, allowed to dry, and sent for electron microscopy imaging at Shenzhen University facilities.

Particle size and zeta potential measurements were carried out using a Zetasizer Lab instrument. UV absorption spectroscopy was conducted with a UV–Vis spectrophotometer, model UV2200. Electron microscopy imaging was performed by Angzhou Yanqu Information Technology Co., Ltd.).

### FCS preparation and nanoparticle fabrication

Perfluoroheptanoic acid (27.5 µmol, 100 mg), EDC (78 mg, 1.5 equivalents), and NH (47.43 mg, 1.5 equivalents) were combined and dissolved in 1 ml of DMSO at room temperature in darkness for 30 min to synthesize activated perfluoroheptanoic acid. CS (200 mg) was dissolved in a 0.5**–**1% acetic acid solution, with the pH adjusted to 6.5. The activated perfluoroheptanoic acid was gradually added dropwise to the CS solution and stirred for 12 h. Post stirring, the mixture was centrifuged at 4000 rpm for 20 min to remove impurities. The supernatant was then purified using a reagent bag (MWCO 3500 Da) in double-distilled water for 48 h, followed by freeze-drying and storage at -20 °C. An aqueous FCS solution (2 mg/ml) was prepared by combining CAST-crRNAa with FCS at mass ratios of 1:2, 1:4, 1:6, and 1:8. Each sample was divided into two groups: one heated at 100 ℃ for 5 min to induce protein denaturation, and the other left untreated. Both groups were subjected to 8% SDS-PAGE at 80 V for 1 h, followed by staining with Coomassie Brilliant Blue for 1 h and destaining for 2 h before imaging.

A CAST-crRNAa:FCS solution (mass ratio 1:4) was prepared, with particle size measured every two days over two weeks, recording the Polydispersity Index (PDI). These samples were used for subsequent particle size and zeta potential determinations.

FCS and BSA@FBZ were prepared according to BSA quantification, with FCS:BSA mass ratios of 4:1, 2:1, 1:1, and 1:2 to formulate BSA@FBZ/FCS. Their particle sizes and zeta potentials were measured.

The solutions of CAST-crRNAa, FCS, and CAST-crRNAa/FCS NPs were analyzed using a UV–visible spectrophotometer. The solutions of BSA@FBZ and BSA@FBZ/FCS NPs were subjected to FTIR analysis.

For release efficiency studies, 0.12 mg/ml BSA@FBZ/FCS was placed in four reagent bags, each containing 4 ml. Each bag was then immersed in 20 ml of a solution at pH 7.4, 6.5, and 5 in 40% acetone, and 10 mmol/L H_2_O_2_ in 0% acetone. A 1 ml sample of the medium solution was extracted at each time point. High-performance liquid chromatography (HPLC) was utilized to detect the concentration of FBZ in the solution using the standard curve. FBZ displayed a UV absorption peak at approximately 254 nm. The standard curve for FBZ was defined as y = 172355x—1939.5. The concentration of FBZ in the medium was calculated based on the area under a specific peak, with release efficiency calculated as the ratio of the FBZ content in the medium to the initial FBZ content.

BSA@FBZ/FCS NPs and CAST-crRNAa/FCS NPs were prepared, then put NPs in the 4, 25, 37 and 70 Degree Celsius water bath respectively for 30 min Then Hydrodynamic diameter and PI were detected by zetasizer.

### Transepithelial delivery across tissues

Six C57L mice were individually inoculated with 30*10^4^ MB49 cells into their bladders and subsequently divided into three groups: BSA, BSA/CS NPs, and BSA/FCS NPs, which received perfusion therapy. Two h post-treatment, the mice were euthanized using carbon dioxide gas, and their bladders were harvested, embedded in a freezing medium, and stored at -20 °C. Using a cryotome pre-cooled to -20 °C, vertical sections of the bladder (10 µm thick) were prepared. These sections were fixed with 4% paraformaldehyde (PFA) for 15 min, washed thrice for 5 min each, stained with DAPI (1:10,000, Thermo Fisher Scientific) for 10 min, and imaged using confocal microscopy.

C57L mice were individually inoculated with 30*10^4^ MB49 cells into their bladders. Mice were treated by Cy5.5-BSA/FCS NPs after 3 days.Fluorescence signal.

were detected by In vivo imaging system at 0-16 h(Aniview100).

Immortalized human ureteric epithelial cells (SV-HUC-1) were uniformly seeded in eight confocal dishes at a density of 6 × 10^5^ cells/dish. Upon reaching 90% confluence, they were divided into CAST/B@F and CAST/B@F/FCS NP groups. The cells were co-incubated with the respective materials for 24 h. Post-incubation, the culture medium was removed, and the cells were washed thrice with PBS. This was followed by fixation in 4% PFA on ice for 15 min, blocking with 2% BSA for 1 h, overnight incubation at 4 °C with primary antibodies against claudins, E-cadherin, MLC, and p-MLC, and three subsequent washes. The cells were then incubated with FITC or Cy5.5 fluorescent secondary antibodies, washed three times, incubated with DAPI, and imaged using confocal microscopy.

### Cellular uptake

The MB49 cells were evenly seeded onto five confocal dishes at a density of 5 × 10^5^ cells/dish. Post cell adherence, they were treated with regular culture medium, CAS-crRNAa, CAST-crRNAa, CAS-crRNAa/FCS NPs, and CAST-crRNAa/FCS NPs. Following a 2-h incubation, the culture medium was removed, and the cells were washed three times with PBS, fixed in 4% PFA on ice for 15 min, washed, permeabilized with 0.1% Triton X-100 (Macklin) for 20 min, washed, blocked with 2% BSA for 1 h, incubated with Cas13a antibody (1:1000, R&D Systems) at room temperature for 3 h, washed three times, incubated with Cy5.5 fluorescent secondary antibodies for 1 h, washed three times, incubated with DAPI, and imaged using confocal microscopy.

Three separate 6-well plates were prepared for assays involving CAST-crRNAa, Cy5.5-BSA/FCS, and Cy5.5-BSA@FBZ/FCS. Each well was seeded with 5 × 10^5^ MB49 cells. Following a 1-h incubation with various agents (chlorpromazine, cytochalasin, genistein, filipin, and wortmannin) in a normal culture medium, CAST-crRNAa, Cy5.5-BSA/FCS NPs, and Cy5.5-BSA@FBZ/FCS NPs were added to each respective plate. Following a 2-h incubation, cells were digested with trypsin, washed three times with PBS, and analyzed using flow cytometry.

### Escape of nanoparticles from lysosomes:

Sixteen confocal dishes were prepared and divided into four groups; each dish was seeded with 6 × 10^5^ MB49 cells. Post-adherence, the cells were treated with Cy5.5-BSA, Cy5.5-BSA/FCS, Cy5.5-BSA@FBZ, or Cy5.5-BSA@FBZ/FCS. Incubation was halted at 0.5, 1, 2, and 4-h intervals. Then, a lysotracker-containing medium was added, and the cells were incubated for an additional h. Following medium removal, careful washing, DAPI staining, and confocal microscopy were performed.

### PDL1 In vitro knockout experiment:

For the MB49 cells seeded in a 4-well (6-well plate) setup, CAST-crRNAa/FCS was prepared at concentrations of 0, 2.5, 5, and 7.5 µg/ml, and the cells were incubated for 16 h. Total RNA was extracted and reverse transcribed into cDNA, and qPCR was performed using GAPDH as an internal reference. Each sample was analyzed in triplicate.

For the MB49 cells seeded in five wells (6-well plate), treatments including PBS, CAS-crRNAa, CAST-crRNAa, CAS-crRNAa/FCS, and CAST-crRNAa/FCS were prepared and incubated with the cells for 16 h. Total RNA was extracted and converted into cDNA, and qPCR was conducted using GAPDH as an internal reference. Each sample was analyzed in triplicate.

For the MB49 cells seeded in five confocal dishes (6 × 10^4^ cells/dish), treatments included PBS, CAS-crRNAa, CAST-crRNAa, CAS-crRNAa/FCS NPs, and CAST-crRNAa/FCS NPs with 5 µg/ml CAST for 24 h. Following the incubation, the culture medium was removed, and cells were washed three times with PBS. The cells were then fixed in 4% PFA on ice for 15 min, washed, permeabilized with Triton X-100, blocked with 2% BSA, incubated with PDL1 primary antibodies overnight at 4 °C, followed by incubation with FITC antibodies, DAPI staining, and confocal microscopy imaging**.**

For the MB49 cells seeded in six confocal dishes (6 × 10^4^ cells/dish), treatments included PBS, BSA@FBZ/FCS NPs, CAST/B@F, CAST-crRNAa/FCS NPs, CAS/B@F/FCS, and CAST/B@F/FCS with 5 µg/ml CAST and 1 µg/mlBSA@FBZ for 24 h. Post-treatment, the culture medium was removed, and cells were washed three times with PBS. The cells were fixed in 4% PFA on ice for 15 min, washed, permeabilized with Triton X-100, blocked with 2% BSA, incubated with PDL1 primary antibodies overnight at 4 °C, followed by incubation with FITC antibodies, DAPI staining, and confocal microscopy imaging**.**

For the MB49 cells seeded in a 6-well plate, following a 24-h incubation with PBS, BSA@FBZ/FCS NPs, CAST/B@F, CAST-crRNAa/FCS NPs, CAS/B@F/FCS, and CAST/B@F/FCS, the culture medium was removed. Cells were digested with trypsin for 1 min, digestion was terminated with culture medium, and the cells were collected, centrifuged, lysed, and subjected to BCA quantification. Following protein quantification, the samples were prepared for SDS-PAGE gel electrophoresis, transferred onto PVDF membranes, blocked with skimmed milk, incubated with PDL1 primary antibodies (1:1000) and TUBULIN mouse primary antibodies (1:1000) overnight at 4 °C. After washing with TBST, secondary antibodies were applied, followed by visualization.

For the MB49 and T24 cells seeded in a 6-well plate respectively, following a 24-h incubation with PBS, BSA@FBZ/FCS NPs, CAST/B@F, CAST-crRNAa/FCS NPs, CAS/B@F/FCS, and CAST/B@F/FCS, the culture medium was removed. Cells were digested with trypsin for 1 min, digestion was terminated with culture medium, and the cells were collected, after washing the cells with PBS for 3 times, the cells were added with PDL1 fluorescent antibody, incubated for 30min, and detected by flow cytometry.

### Cell proliferation and apoptosis:

A 96-well plate with 24 wells was seeded with 1000 cells/well and divided into six groups: PBS, BSA@FBZ/FCS, CAST/B@F, CAST-crRNAa/FCS, CAS/B@F/FCS, and CAST/B@F/FCS. Following overnight incubation, the culture medium was replaced with a medium containing Rhodamine B for 1 h. Absorbance was measured at approximately 565 nm.

In a 6-well plate, 6 × 10^4^ cells/well were seeded. Following overnight incubation with PBS, BSA@FBZ/FCS, CAST/B@F, CAST-crRNAa/FCS, CAS/B@F/FCS, or CAST/B@F/FCS, apoptosis was detected using an apoptosis detection kit.

### FBZ regulatory mechanism

In a 6-well plate with approximately 6 × 10^5^ cells/well, treatments including BSA@FBZ/FCS, BSA@FBZ, FBZ, and PBS were administered. Following a 16-h incubation, total RNA was extracted and reverse-transcribed into cDNA, and qPCR was performed using GAPDH as an internal reference. Each sample was analyzed in triplicate. Another 6-well plate (approximately 6 × 10^5^ cells/well) was treated with BSA@FBZ/FCS, BSA@FBZ, FBZ, FCS, or PBS for 24 h. Trypsin digestion was conducted for 1 min post-treatment and terminated with the medium. Cells were collected, lysed, quantified using BCA, prepared for SDS-PAGE gel electrophoresis, transferred onto PVDF membranes, blocked with skimmed milk, and incubated with primary antibodies against HK2, NF-κB, p-IKBa, PDL1, and GAPDH (1:1000) overnight at 4 °C. After washing with TBST, secondary antibodies were applied, followed by visualization.

### CAST/B@F/FCS in vivo treatment:

Forty-one C57L mice were divided into six groups: PBS, BSA@FBZ/FCS, CAST/B@F, CAST-crRNAa/FCS, CAS/B@F/FCS, and CAST/B@F/FCS. On DAY0, mice were injected with 3 × 10^4^ cells into the bladder wall. Starting from DAY3, mice underwent perfusion therapy with doses of CAST-crRNAa (2 mg/kg) and BSA@FBZ (0.36 mg/kg) every three days for five consecutive days. Observations concluded on DAY32, with bioluminescence imaging conducted weekly starting from DAY6, and fluorescence data recorded. The survival status, including body weight, was recorded daily.

Additionally, 18 mice were divided into six groups and subjected to the same tumor inoculation method. Three days after the second treatment, the mice were euthanized using carbon dioxide gas anesthesia, and tissues, including tumors, heart, liver, spleen, lungs, and kidneys, were collected. Histological sections were prepared for H&E staining (heart, liver, spleen, lungs, and kidneys) and Tumors for TUNEL staining. Immunohistochemical assays were performed using PDL1 and KI67 markers.

### Flow cytometry analysis of tumor PDL1 and immune cells:

The tumor tissues from the remaining 18 mice were enzymatically digested and filtered. The cells were processed for flow cytometric analysis to detect PDL1, CD8 + /CD4 + T cells, DCs, Treg cells, MDSCs, TNF-a, IFN-y and M1/M2 macrophages using specific fluorescent antibodies (approximately 10 × 10^5^ cells/sample). Flow cytometry was performed after incubating the cells with the respective antibodies.

### Supplementary Information

Supplementary Material 1.

## Data Availability

The datasets used and/or analysed during the current study are available from the corresponding author upon reasonable request.

## Abbreviations

CAST: Cas13a-TAT
crRNAa: CRIPSR RNA
PDL1: Programmed Death Ligand 1
FCS: Fluorinated Chitosan
FBZ: Flubendazole
BCG: Bacillus Calmette Guerin
BCa: Bladder cancer
HPLC: High Performance Liquid Chromatography
TEM: Transmission Electron Microscope
FTIR: Fourier transform infrared spectroscopy
MLC: Myosin light chain
DCs: Dendritic cells
Qpcr: Quantitative Real-time PCR
MDSCs: Myeloid-Derived Suppressor Cells
CLSM: Confocal Laser Scanning Microscope
IHC: Immunohistochemistry
