# Supplementary material for: Synergistic intravesical instillation for bladder cancer: CRISPR-Cas13a and fenbendazole combination therapy
Source: J Exp Clin Cancer Res. 2024 Aug 12;43:223. doi: 10.1186/s13046-024-03146-0 (PMC11318243; doi:10.1186/s13046-024-03146-0)
Supplement: Supplementary file 1 — Supplementary Material 1. [file 13046_2024_3146_MOESM1_ESM.docx]

**Supporting information**

**Synergistic Intravesical Instillation for Bladder Cancer: CRISPR-Cas13a and Fenbendazole Combination Therapy**

Mingkang Liang^1,2#^, Yongqiang Wang^3#^, Lisha Liu^2^, Dashi Deng^2^, Yan Zeqin^3^, Lida Feng^2^, Chenchen Li^2^, Yuqing Li^1,3^*, Guangzhi Li^1,2^*

1. Luohu clinical college of Shantou University Medical College, Shantou University Medical College, Shantou, 515000, Guangdong, China

2. Institute of Urology, The Affiliated Luohu Hospital of Shenzhen University, Shenzhen University, Shenzhen, 518000, China

3. Department of Urology, South China Hospital, Health Science Center, Shenzhen University, Shenzhen, 518116, China

^*^Correspondence: guangzhili@126.com

^#^These authors contributed equally to this work.

KEYWORD: CRISPR-Cas13a， Flubendazole， Fluorinated Chitosan， Bladder cancer， Programmed Death Ligand 1，Transmembrane peptides

**
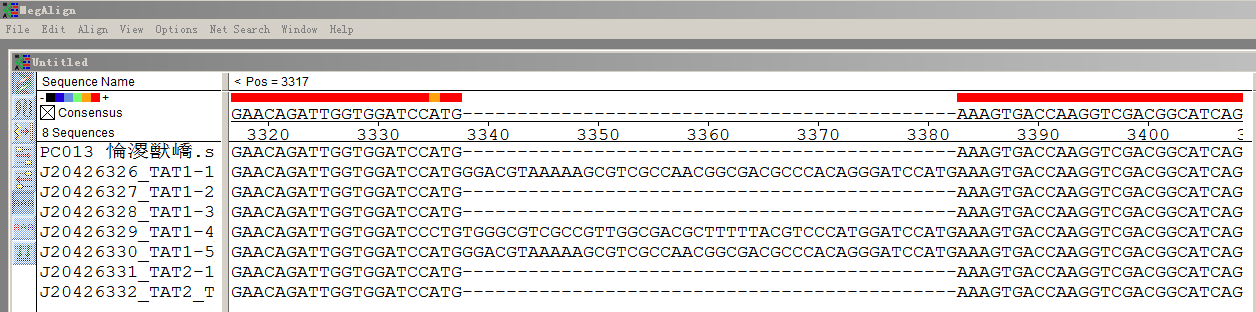
**

**FigureS1** Sequencing result

**
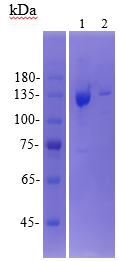
**

**FigureS2** Cas13a-TAT electrophoresis results

**
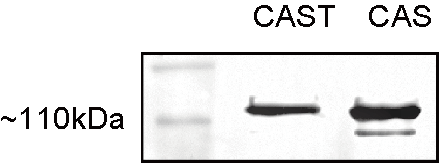
**

**FigureS3** CAST and CAS western blot assay

**
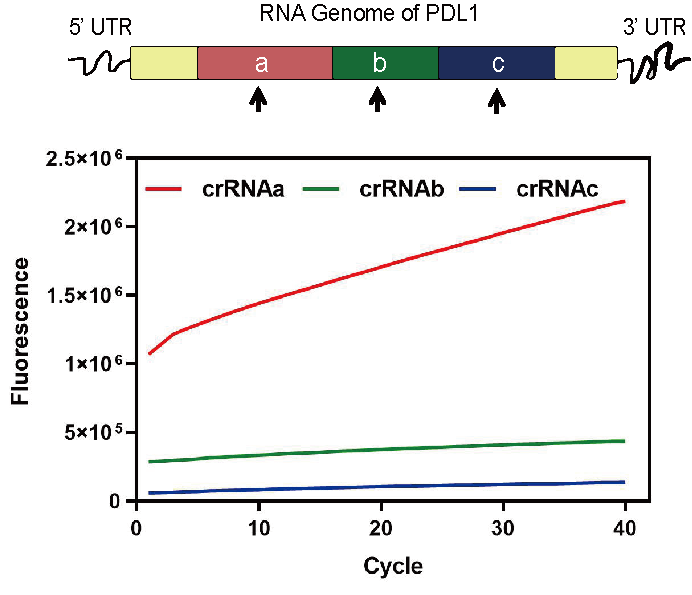
**

**FigureS4** Taqman test results

**
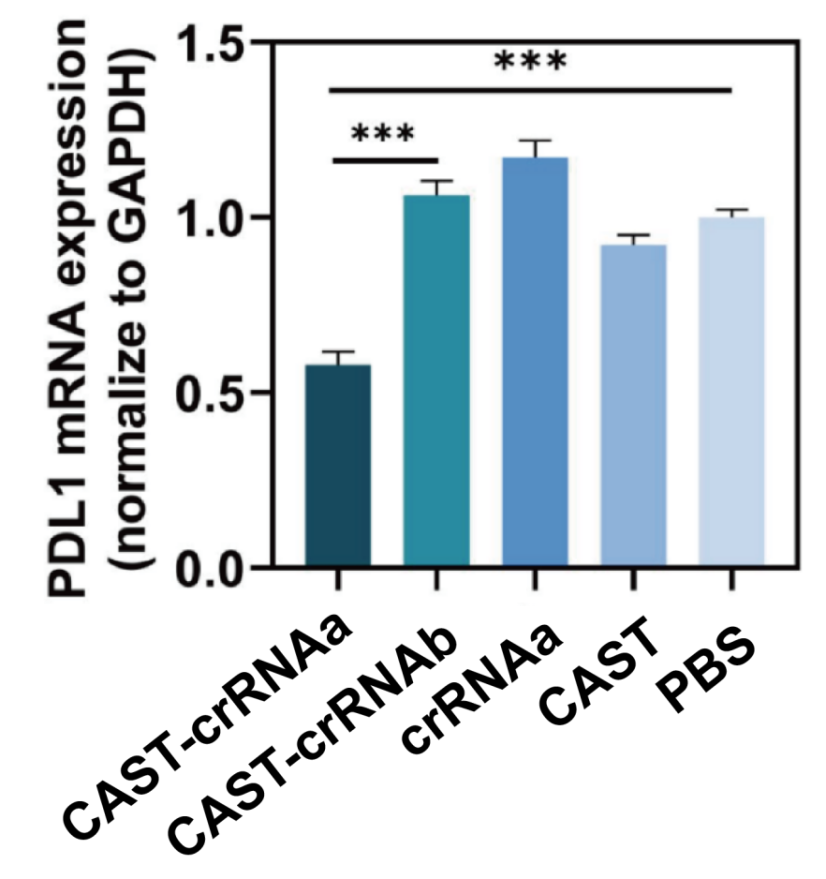
**

**FigureS5** Qpcr result

**
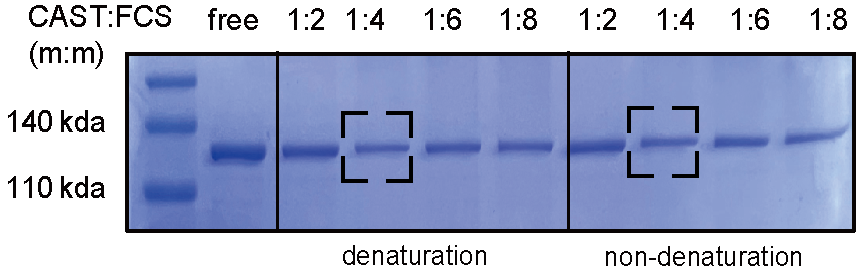
**

**FigureS6** Electrophoretic Coomassie brilliant blue staining in CAST-crRNAa and FCS ratios

**
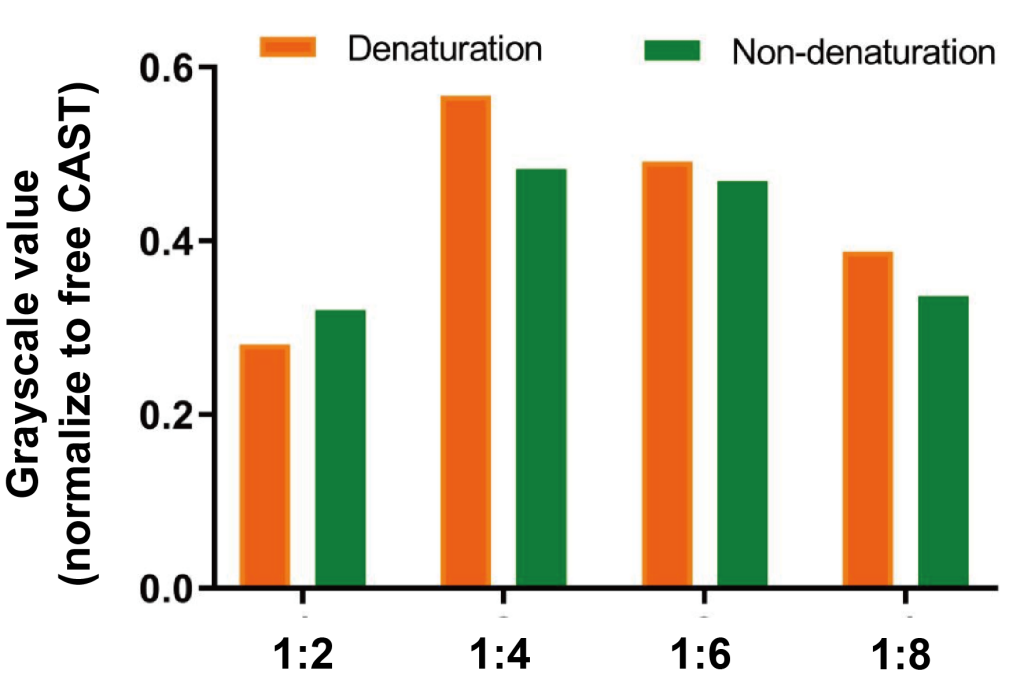
**

**FigureS7** Grayscale statisitics

**
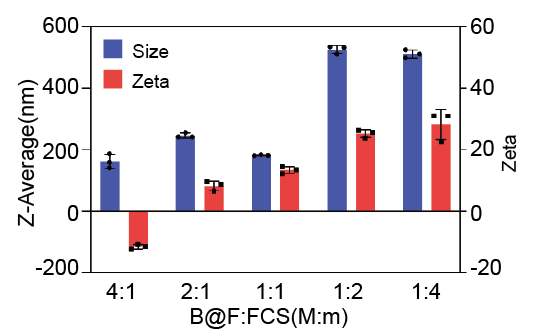
**

**FCS:BSA@FBZ(M:m)**

**FigureS8** Particle size and potential at each ratio of BSA@FBZ and FCS

**
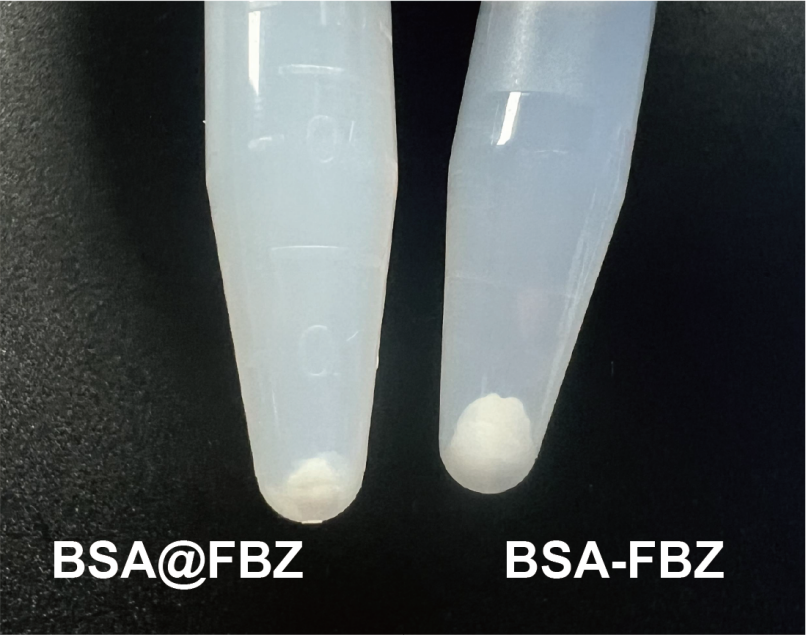
**

**FigureS9** Contrast after impurity removal BSA@FBZand BSA-FBZ

**
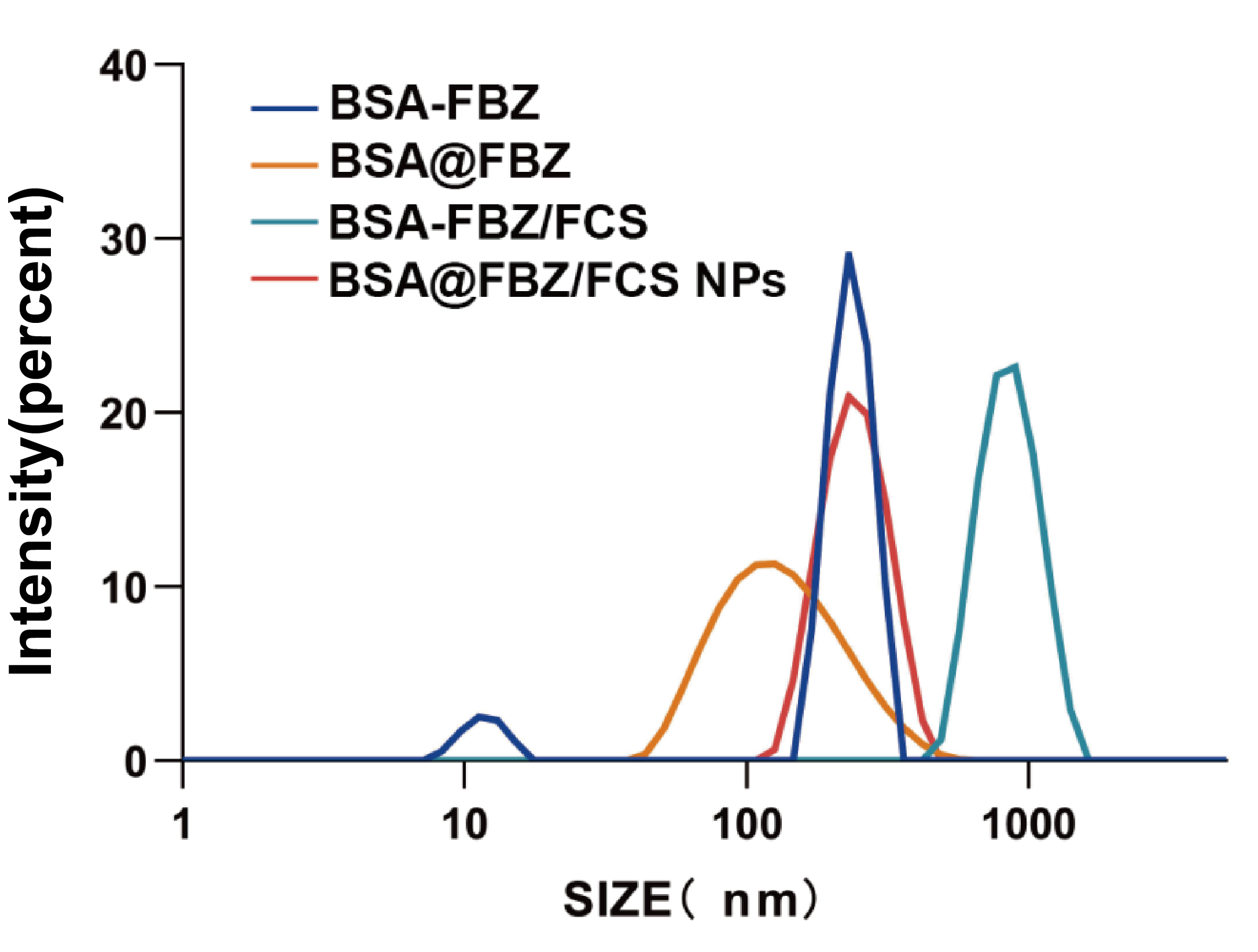
**

**FigureS10** Hydrodynamic diameter of each formula

**
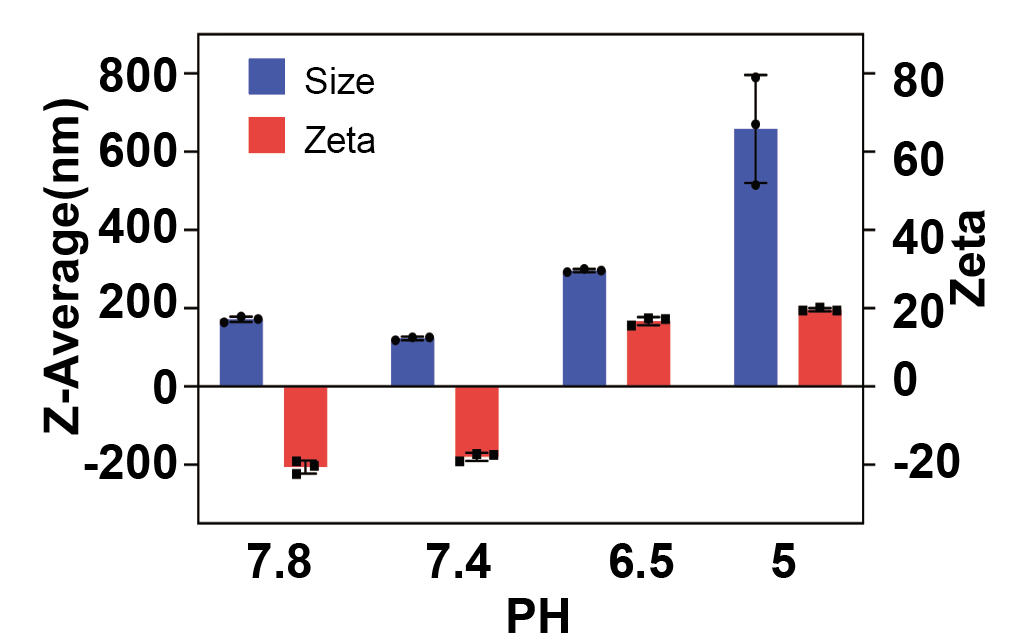
**

**FigureS11** Hydrodynamic diameter and potentiogram of BSA@FBZ at different PH

**
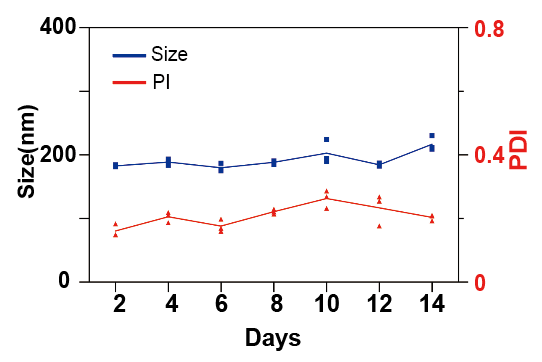
**

**FigureS12** BSA@FBZ's 14-day hydrodynamic diameter


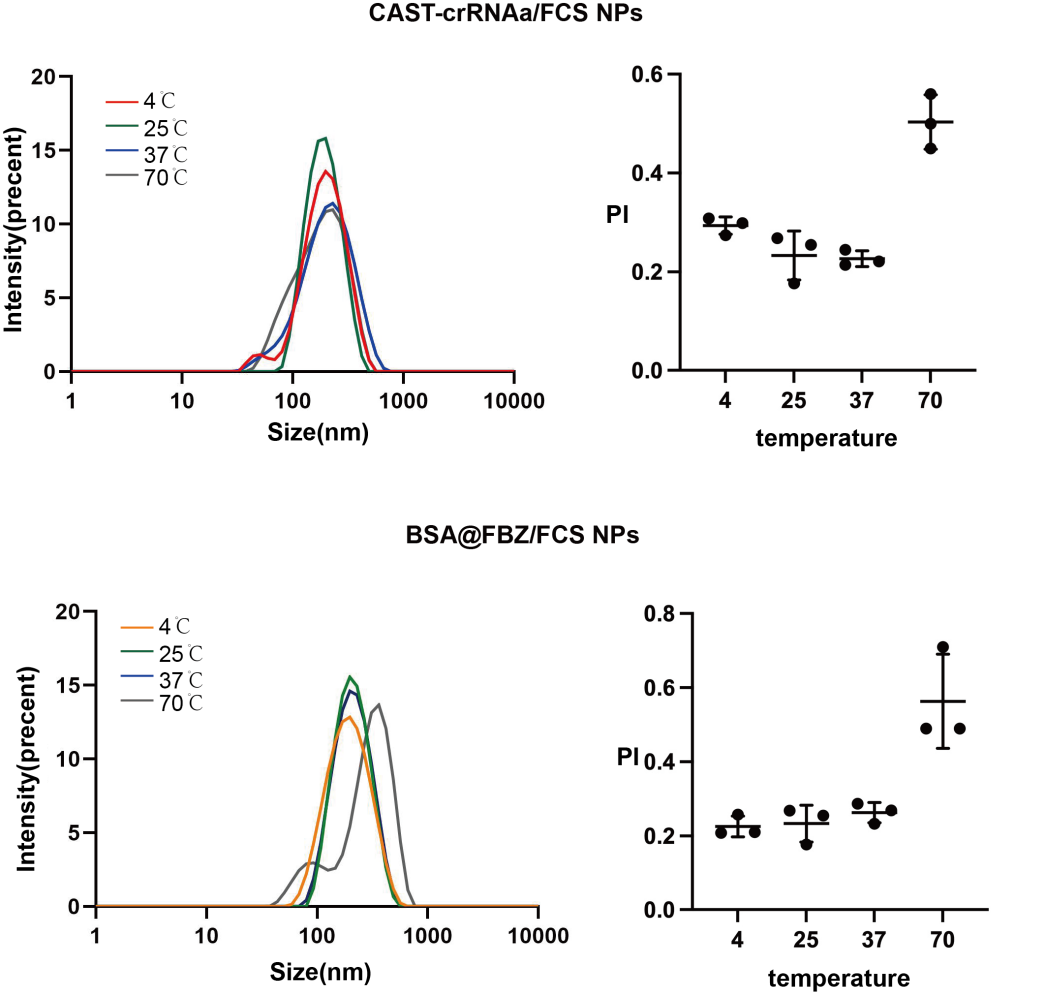


**Figure S13** Hydrodynamic diameter and PI of NPs at Different temperature


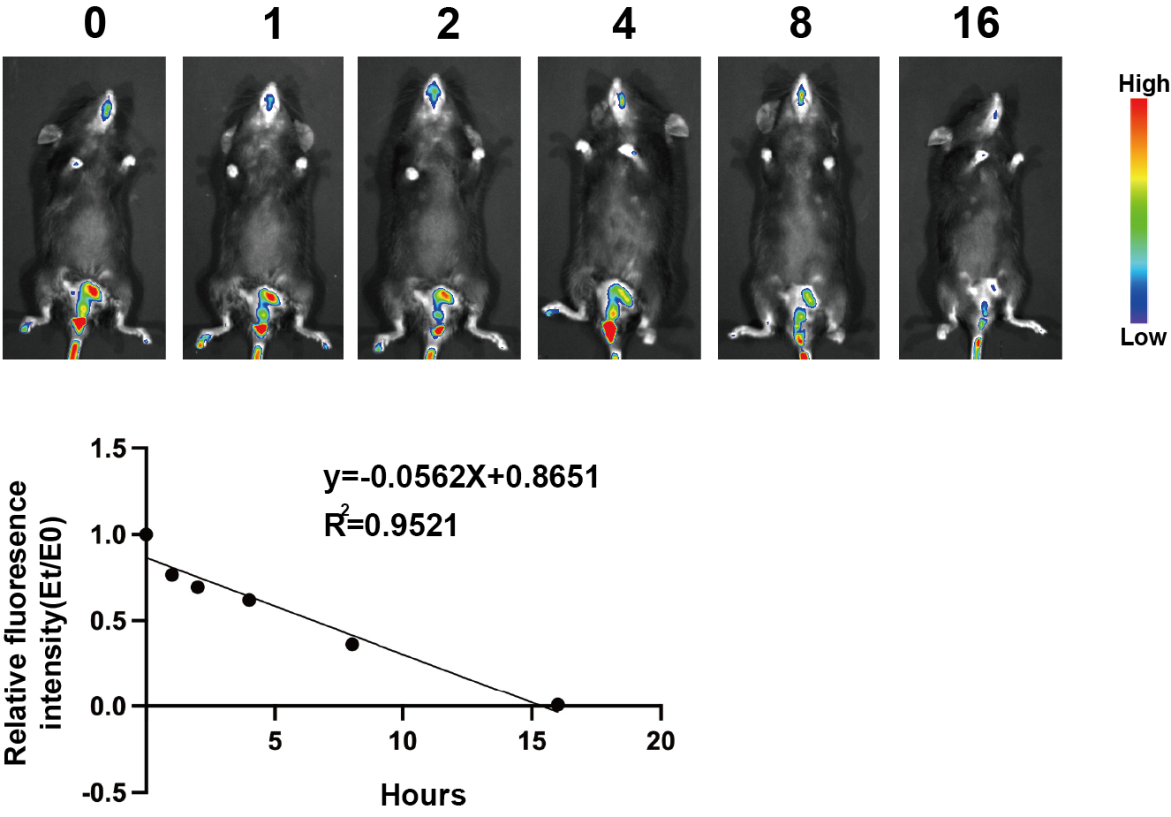


**Figure S14** Half-life of BSA/FCS NPs in bladder

**
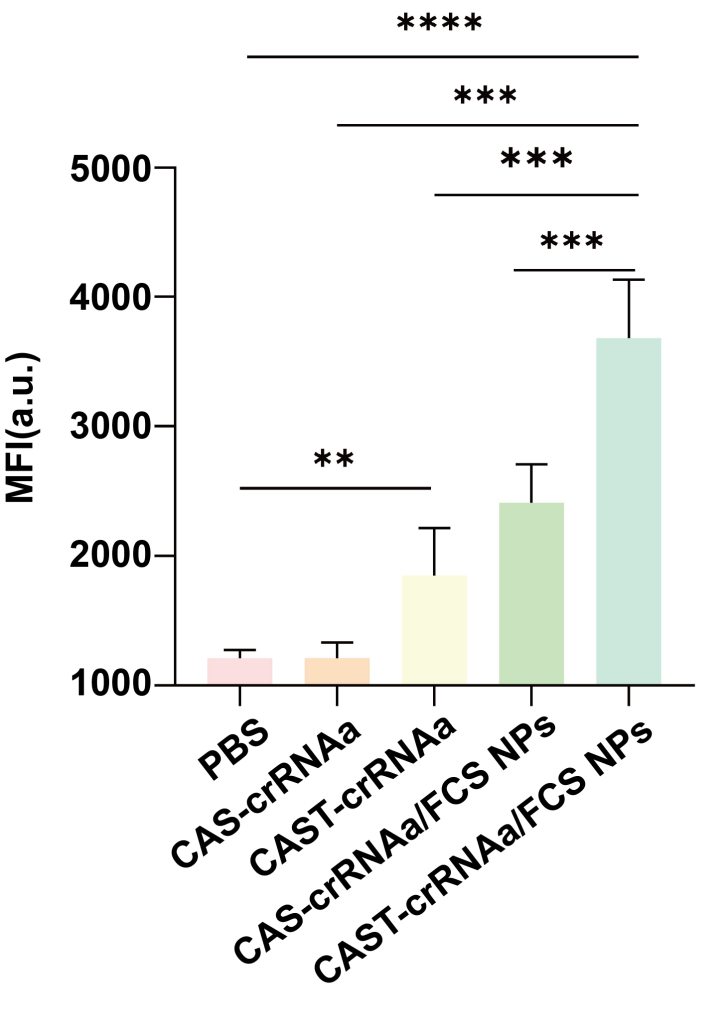
**

**FigureS15** Statistical analysis of MFI value in bladder of CAS and CAST

**
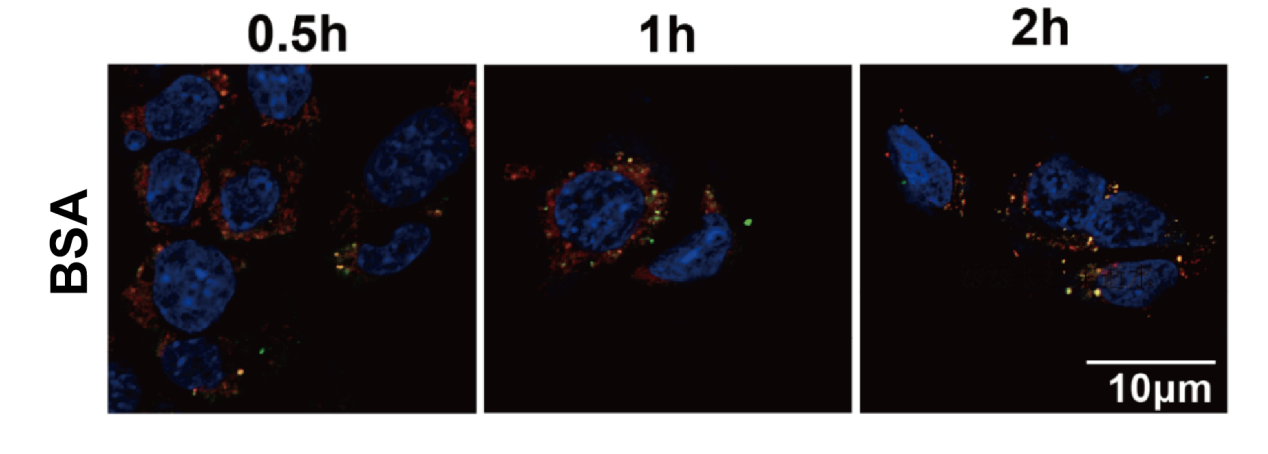
**

**FigureS16** Intracellular fluorescence location of BSA

**
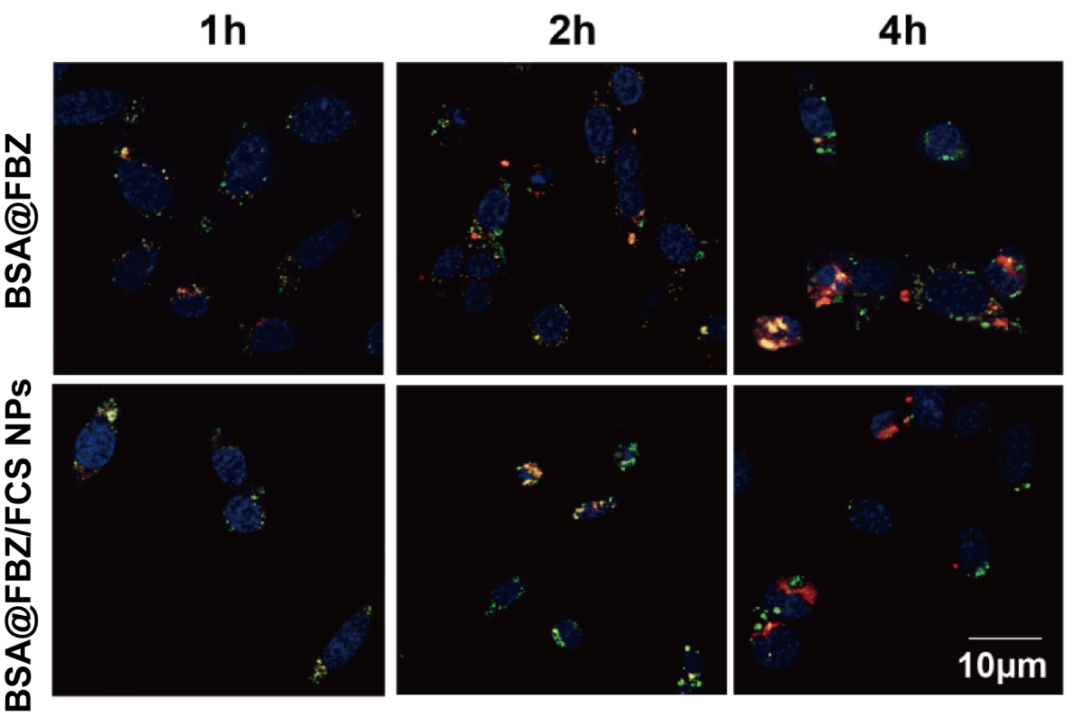
**

**FigureS17** Intracellular fluorescence location of BSA@FBZ


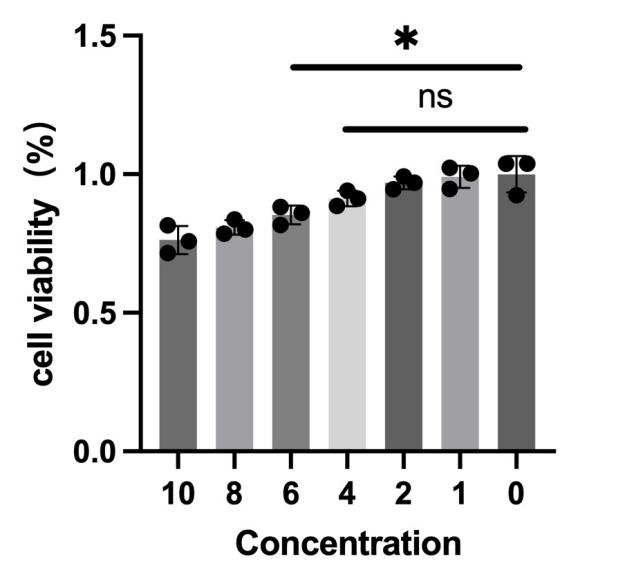

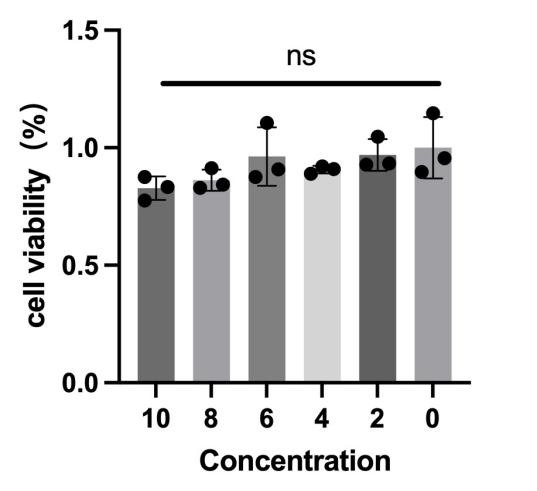


**Figure S18** Cell viability of MB49 andSV-HUC1 treated by different concetrations of CAST-crRNAa/FCS NPs

**
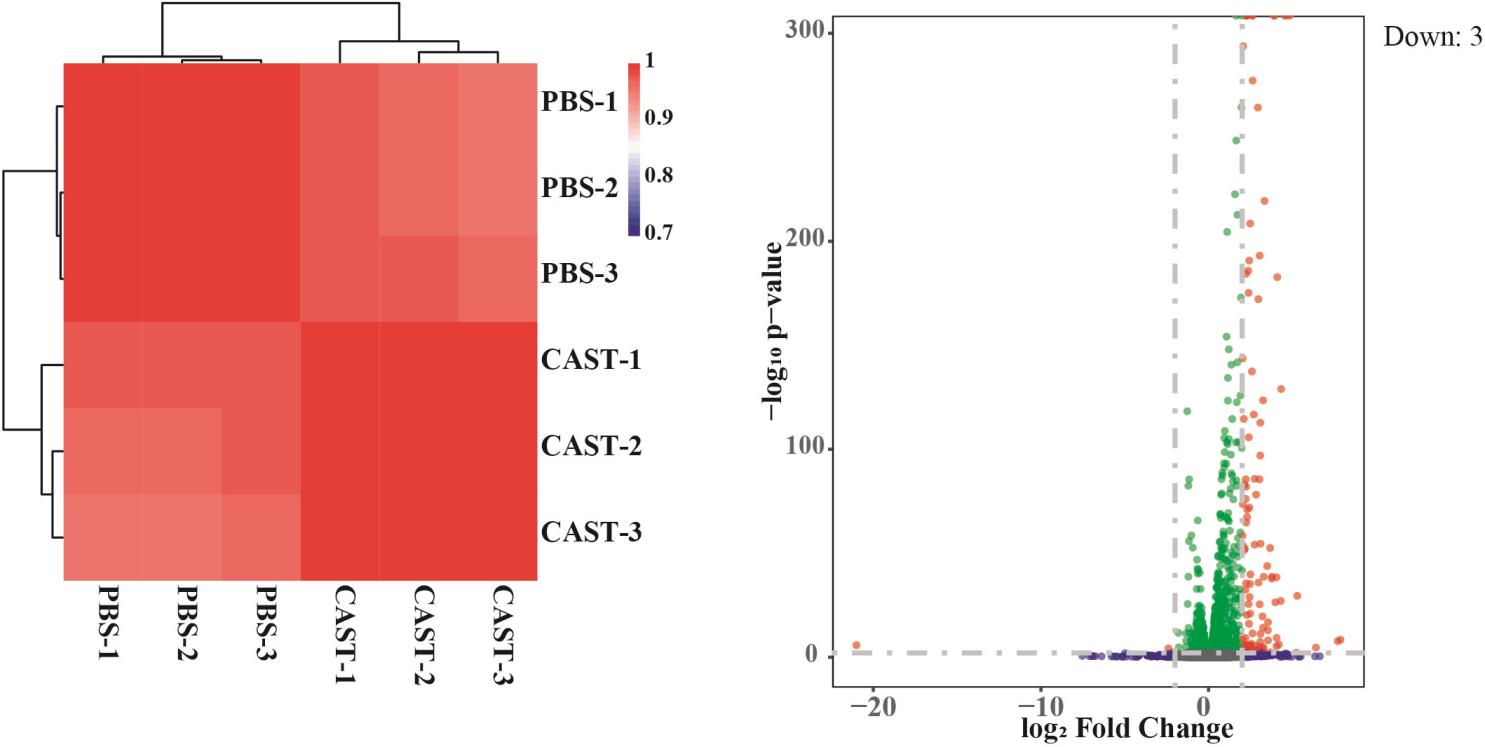
**

**Figure S19** The significantly down-regulated genes of cells treated with CAST-crRNAa/FCS NPs


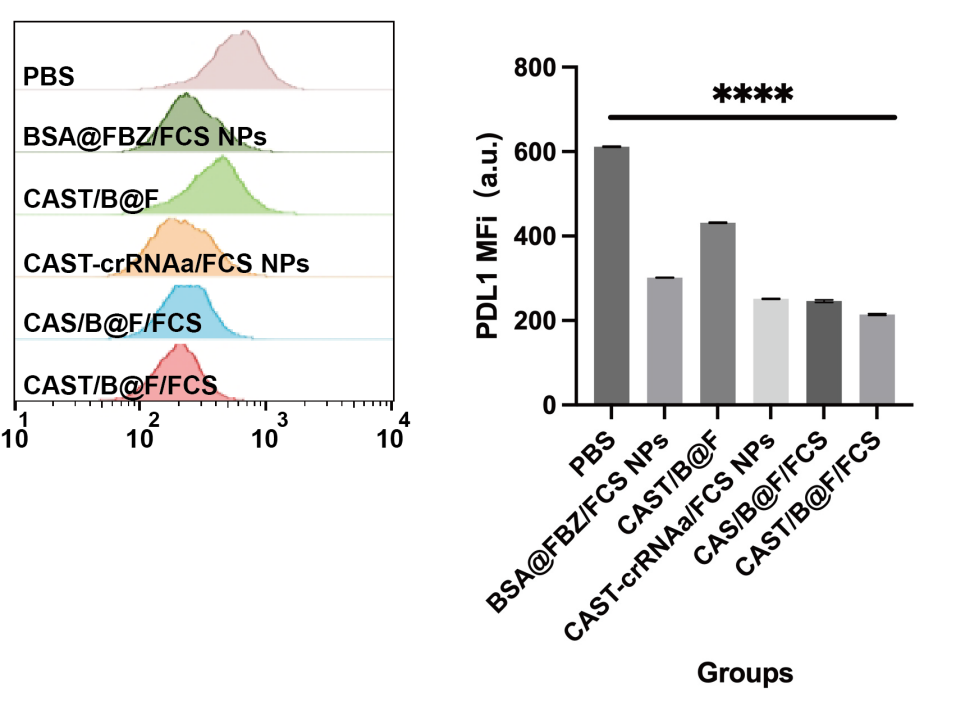


**Figure S20** Flow cytometer analysis of PDL1 expression in MB49


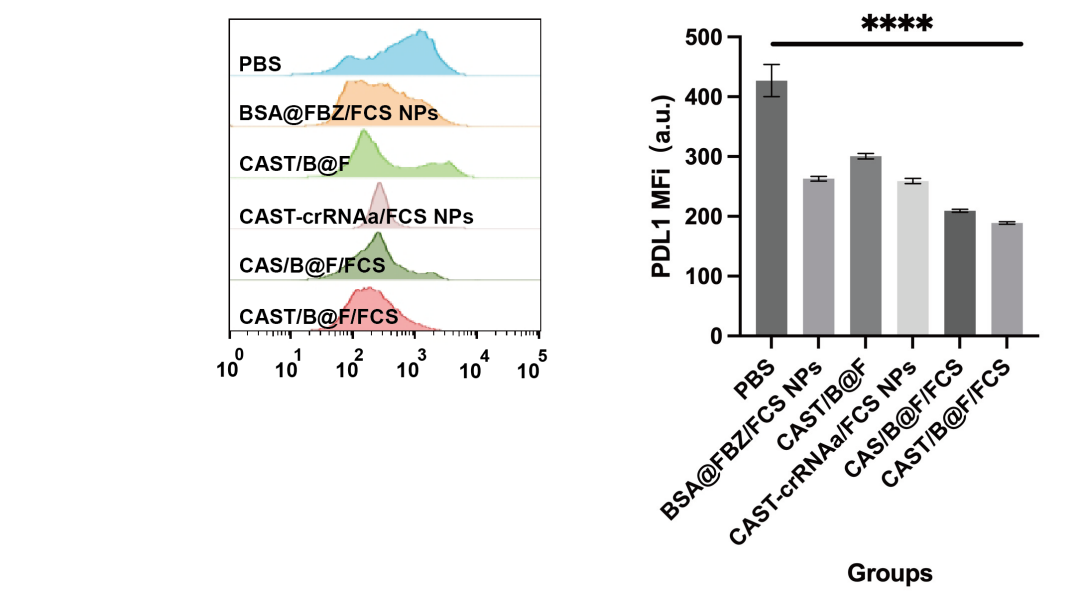


**Figure S21** Flow cytometer analysis of PDL1 expression in T24


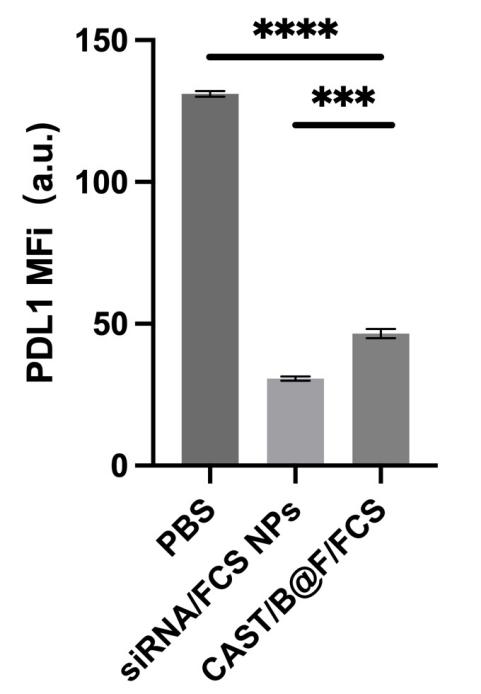


**Figure S22**Flow cytometer analysis of PDL1 expression of siRNA/FCS NPs group in MB49

**
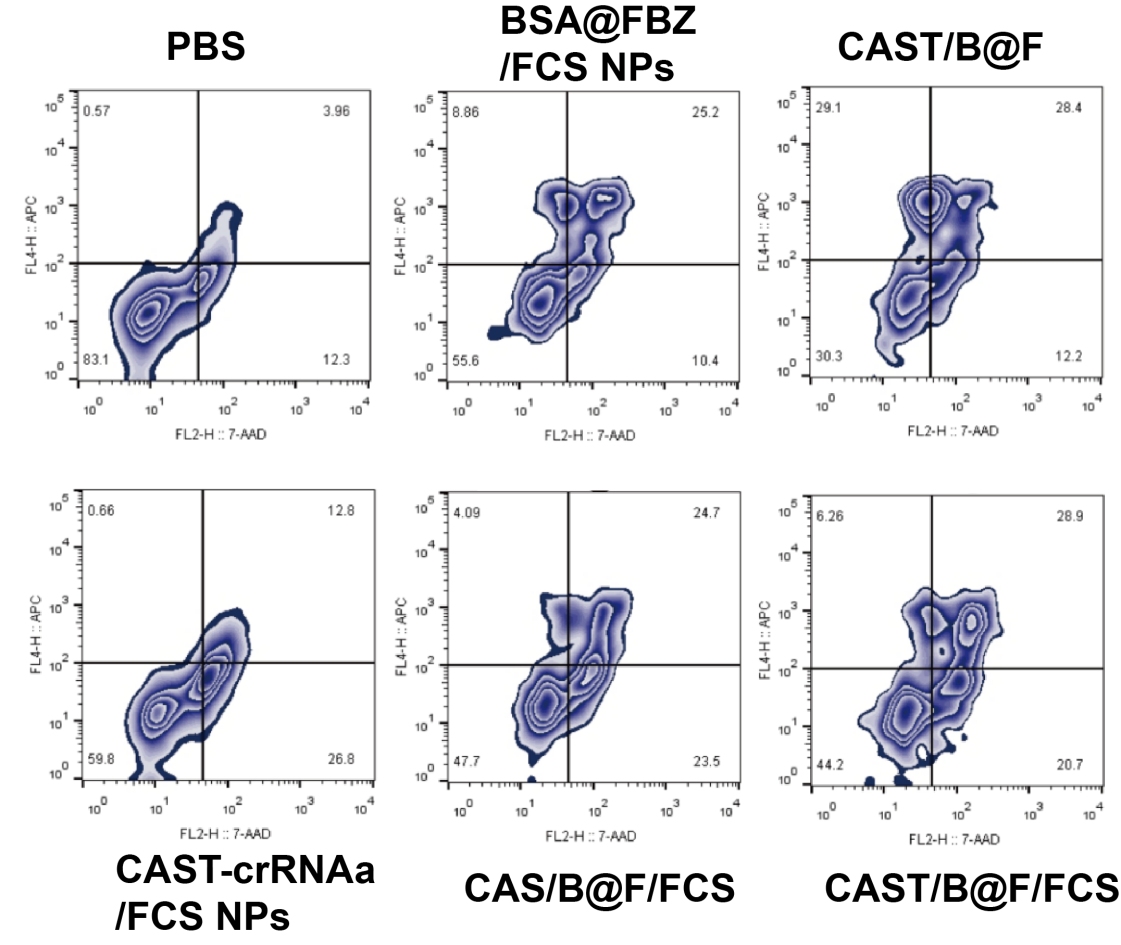
**

**FigureS23** Cell apoptosis in different groups


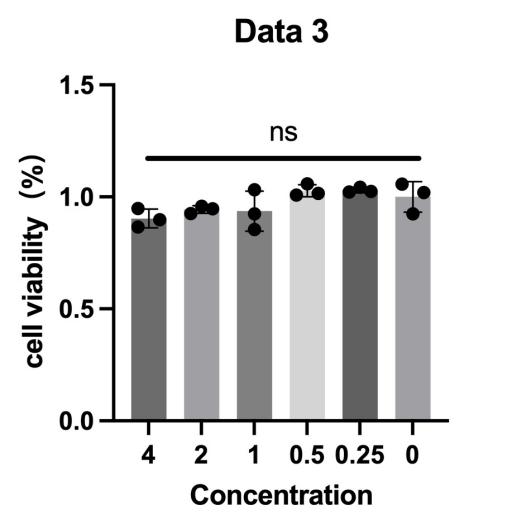


**Figure S24** Cell viability of SV-HUC1 treated by BSA@FBZ/FCS NPs

**
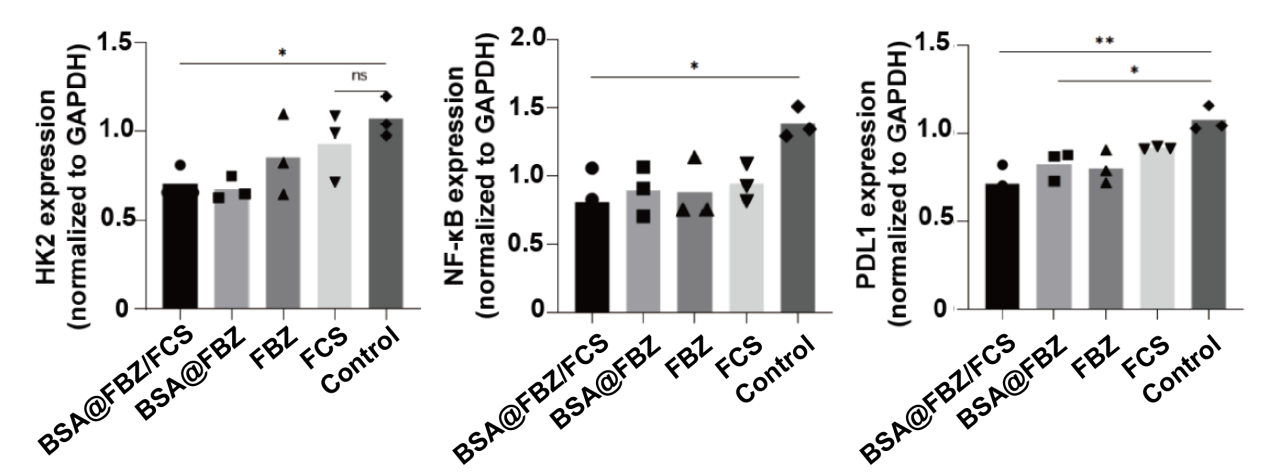
**

**FigureS25** Statistics of grayscale value of western blot


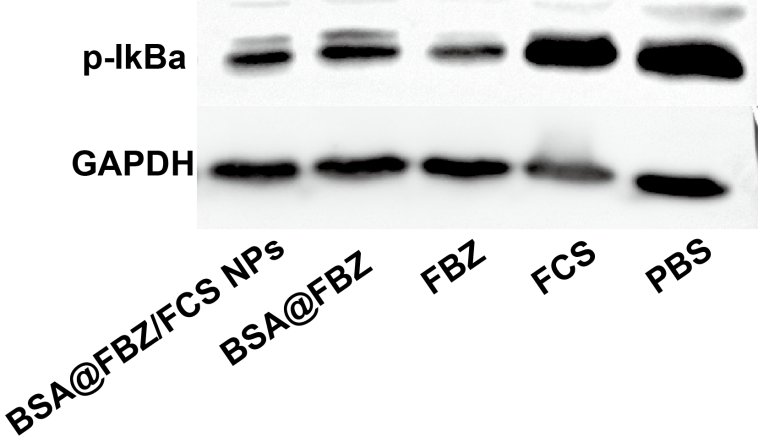


**Figure S26** Western blot analysis of IKB-a


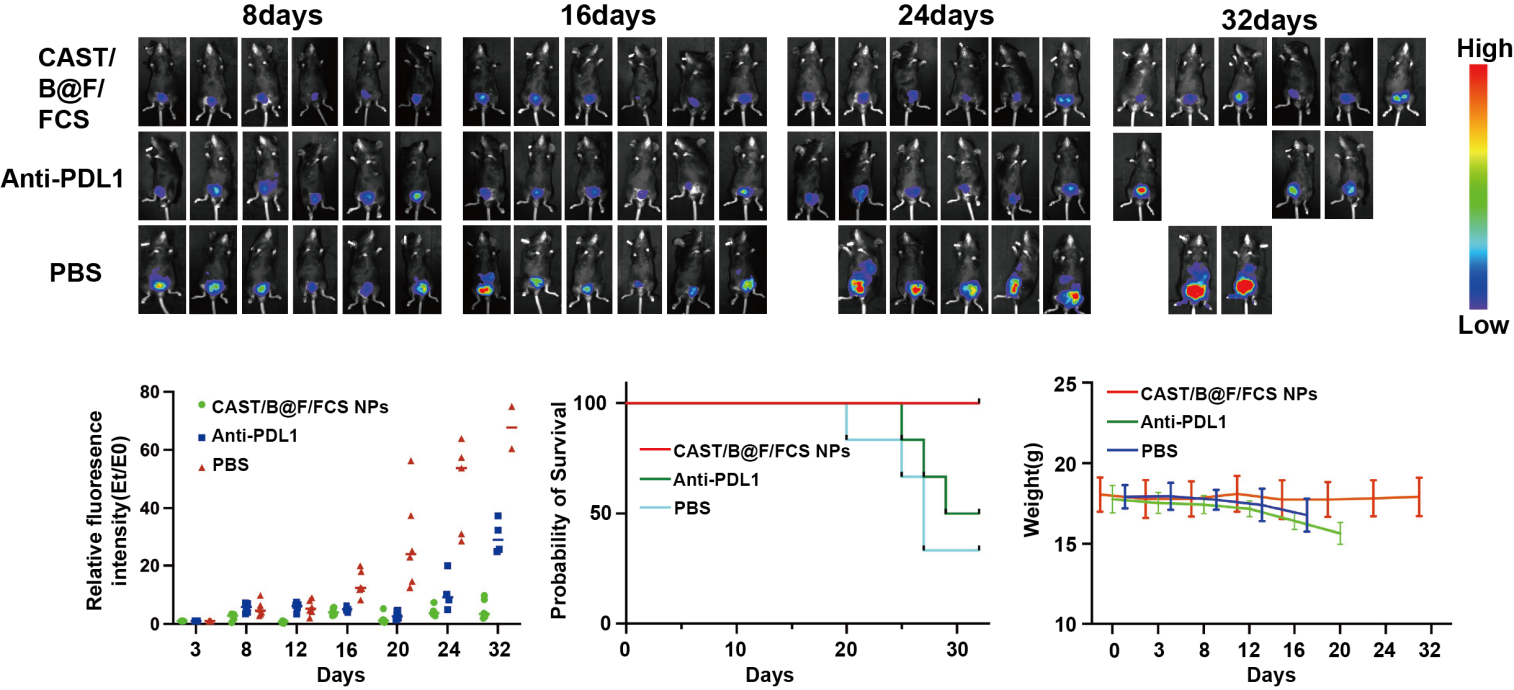


**Figure S27** Fluroscence analysis, survival, Weight of CAST/B@F/FCS, Anti-PDL1,PBS groups

**
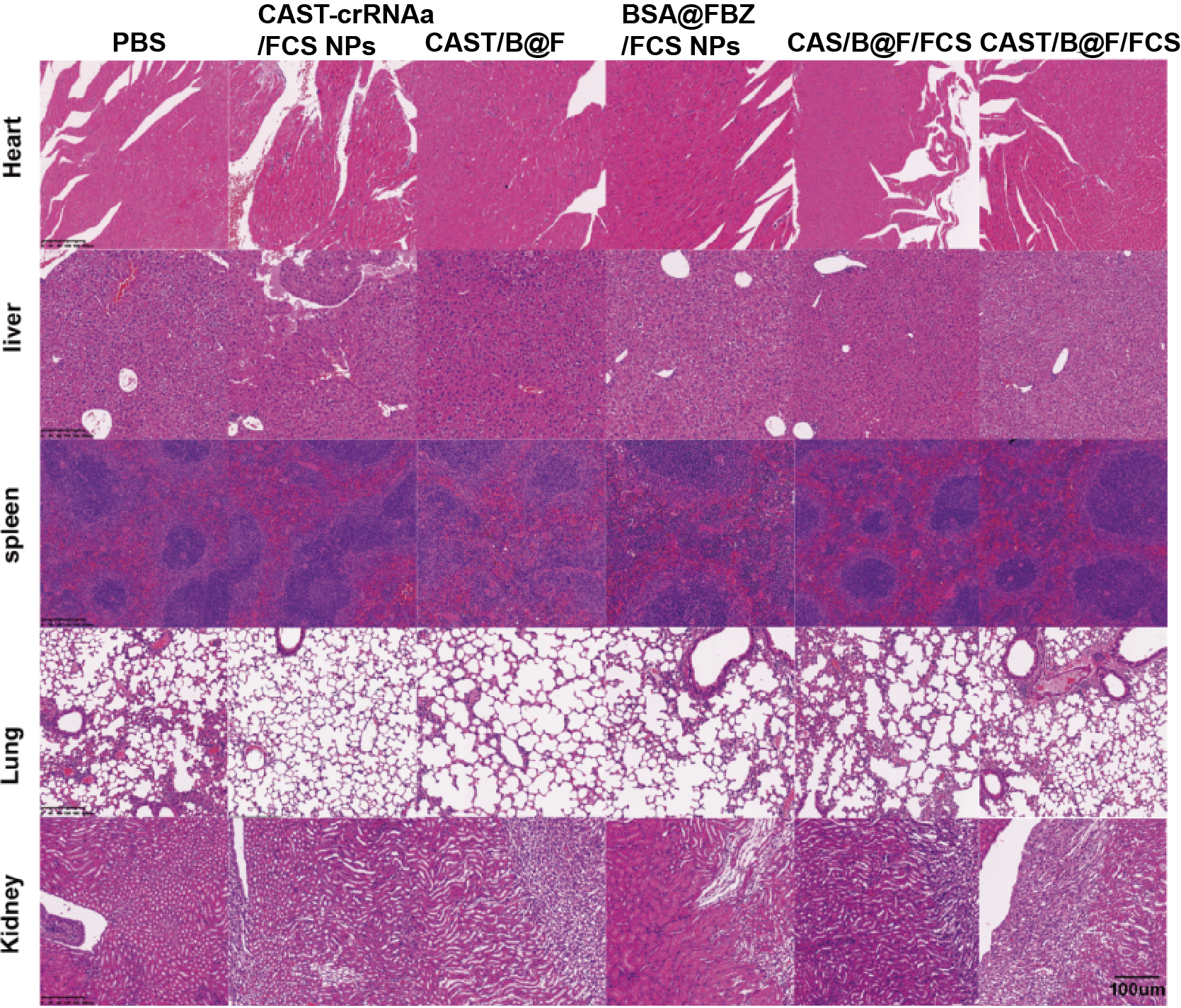
**

**FigureS28** Vital organs H&E staining


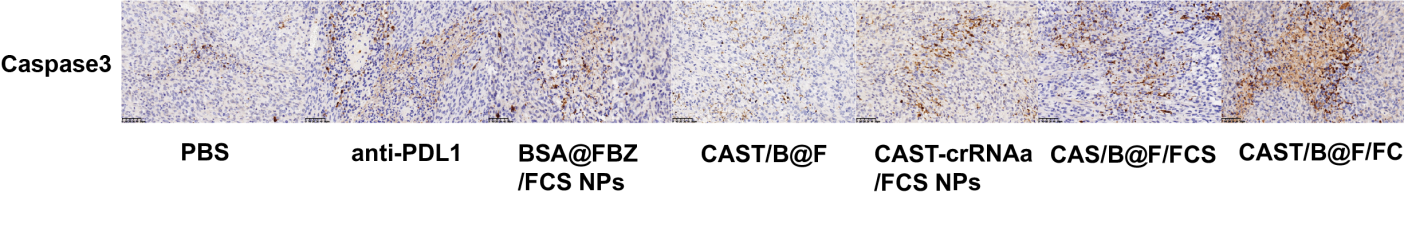


**Figure S29** IHC of Caspase3


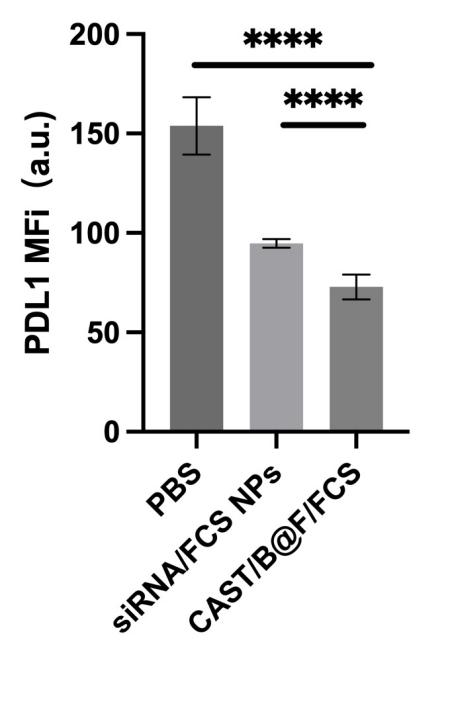


**Figure S30** Flow cytometer analysis of PDL1 expression of siRNA/FCS NPs group in tumors


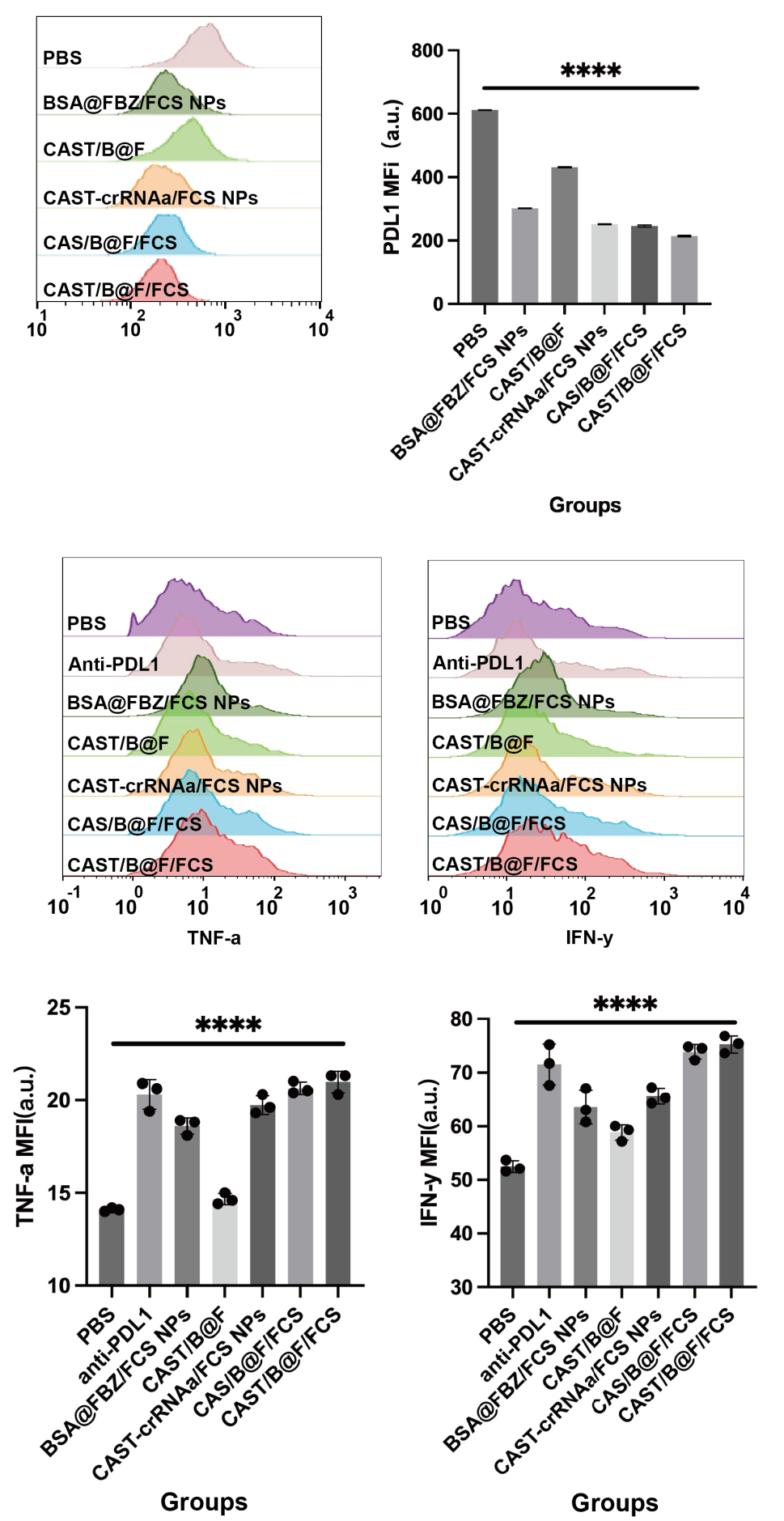


**Figure S31** Flow cytometer analysis of TNF-a and IFN-y expression in tumors
